# The Role of Genetic Polymorphisms in High-Dose Methotrexate Toxicity and Response in Hematological Malignancies: A Systematic Review and Meta-Analysis

**DOI:** 10.3389/fphar.2021.757464

**Published:** 2021-10-21

**Authors:** Zaiwei Song, Yang Hu, Shuang Liu, Dan Jiang, Zhanmiao Yi, Mason M. Benjamin, Rongsheng Zhao

**Affiliations:** ^1^ Department of Pharmacy, Peking University Third Hospital, Beijing, China; ^2^ Institute for Drug Evaluation, Peking University Health Science Center, Beijing, China; ^3^ Therapeutic Drug Monitoring and Clinical Toxicology Center, Peking University, Beijing, China; ^4^ Department of Pharmacy Administration and Clinical Pharmacy, School of Pharmaceutical Sciences, Peking University, Beijing, China; ^5^ Department of Pharmaceutical Sciences, College of Pharmacy, University of Michigan, Ann Arbor, MI, United States

**Keywords:** methotrexate, pharmacogenetics, polymorphism, toxicity, hematological malignancies

## Abstract

**Objective:** High-dose methotrexate (HDMTX) is a mainstay therapeutic agent for the treatment of diverse hematological malignancies, and it plays a significant role in interindividual variability regarding the pharmacokinetics and toxicity. The genetic association of HDMTX has been widely investigated, but the conflicting results have complicated the clinical utility. Therefore, this systematic review aims to determine the role of gene variants within the HDMTX pathway and to fill the gap between knowledge and clinical practice.

**Methods:** Databases including EMBASE, PubMed, Cochrane Central Register of Controlled Trials (CENTRAL), and the Clinical Trials.gov were searched from inception to November 2020. We included twelve single-nucleotide polymorphisms (SNPs) within the HDMTX pathway, involving *RFC1*, *SLCO1B1*, *ABCB1*, *FPGS*, *GGH*, *MTHFR*, *DHFR*, *TYMS*, and *ATIC*. Meta-analysis was conducted by using Cochrane Collaboration Review Manager software 5.3. The odds ratios (ORs) or hazard ratios (HRs) with 95% confidence interval (95% CI) were analyzed to evaluate the associations between SNPs and clinical outcomes. This study was performed according to the PRISMA guideline.

**Results:** In total, 34 studies with 4102 subjects were identified for the association analysis. Nine SNPs involving *MTHFR*, *RFC1*, *ABCB1*, *SLCO1B1*, *TYMS*, *FPGS*, and *ATIC* genes were investigated, while none of studies reported the polymorphisms of *GGH* and *DHFR* yet. Two SNPs were statistically associated with the increased risk of HDMTX toxicity: *MTHFR 677C>T* and hepatotoxicity (dominant, OR=1.52, 95% CI=1.03-2.23; recessive, OR=1.68, 95% CI=1.10–2.55; allelic, OR=1.41, 95% CI=1.01–1.97), mucositis (dominant, OR=2.11, 95% CI=1.31–3.41; allelic, OR=1.91, 95% CI=1.28–2.85), and renal toxicity (recessive, OR=3.54, 95% CI=1.81–6.90; allelic, OR=1.89, 95% CI=1.18–3.02); *ABCB1 3435C>T* and hepatotoxicity (dominant, OR=3.80, 95% CI=1.68-8.61), whereas a tendency toward the decreased risk of HDMTX toxicity was present in three SNPs: *TYMS 2R>3R* and mucositis (dominant, OR=0.66, 95% CI=0.47–0.94); *RFC1 80A>G* and hepatotoxicity (recessive, OR=0.35, 95% CI=0.16–0.76); and *MTHFR 1298A>C* and renal toxicity (allelic, OR=0.41, 95% CI=0.18–0.97). Since the data of prognosis outcomes was substantially lacking, current studies were underpowered to investigate the genetic association.

**Conclusions:** We conclude that genotyping of *MTHFR* and/or *ABCB1* polymorphisms prior to treatment, *MTHFR 677C>T* particularly, is likely to be potentially useful with the aim of tailoring HDMTX therapy and thus reducing toxicity in patients with hematological malignancies.

## Introduction

Acute lymphoblastic leukemia (ALL) is the most common neoplasm in children, accounting for about 30 percent of all pediatric malignancies (Coluzzi et al., 2020). High-dose methotrexate (HDMTX) is commonly defined as an intravenous dose greater than 500 mg/m^2^ (Howard et al., 2016), and HDMTX is recommended as an essential component of chemotherapy for ALL and non-Hodgkin lymphoma (NHL) in clinical guidelines (National Comprehensive Cancer Network, 2021a; b; c). Although breakthroughs have been made in the complex treatment of hematological malignancies, HDMTX still plays a key role and is established as the first-line drug (Gervasini and Mota-Zamorano, 2019). However, patients differ largely in their response to treatment regarding HDMTX pharmacokinetics and toxicities, even when given the identical dose (Schmiegelow, 2009; Giletti and Esperon, 2018). Serious and life-threatening toxicity can occur in patients, leading to treatment interruption and discontinuation, dose reduction, poor prognosis, and even death (Howard et al., 2016; Purkayastha et al., 2018).

**FIGURE 1:**
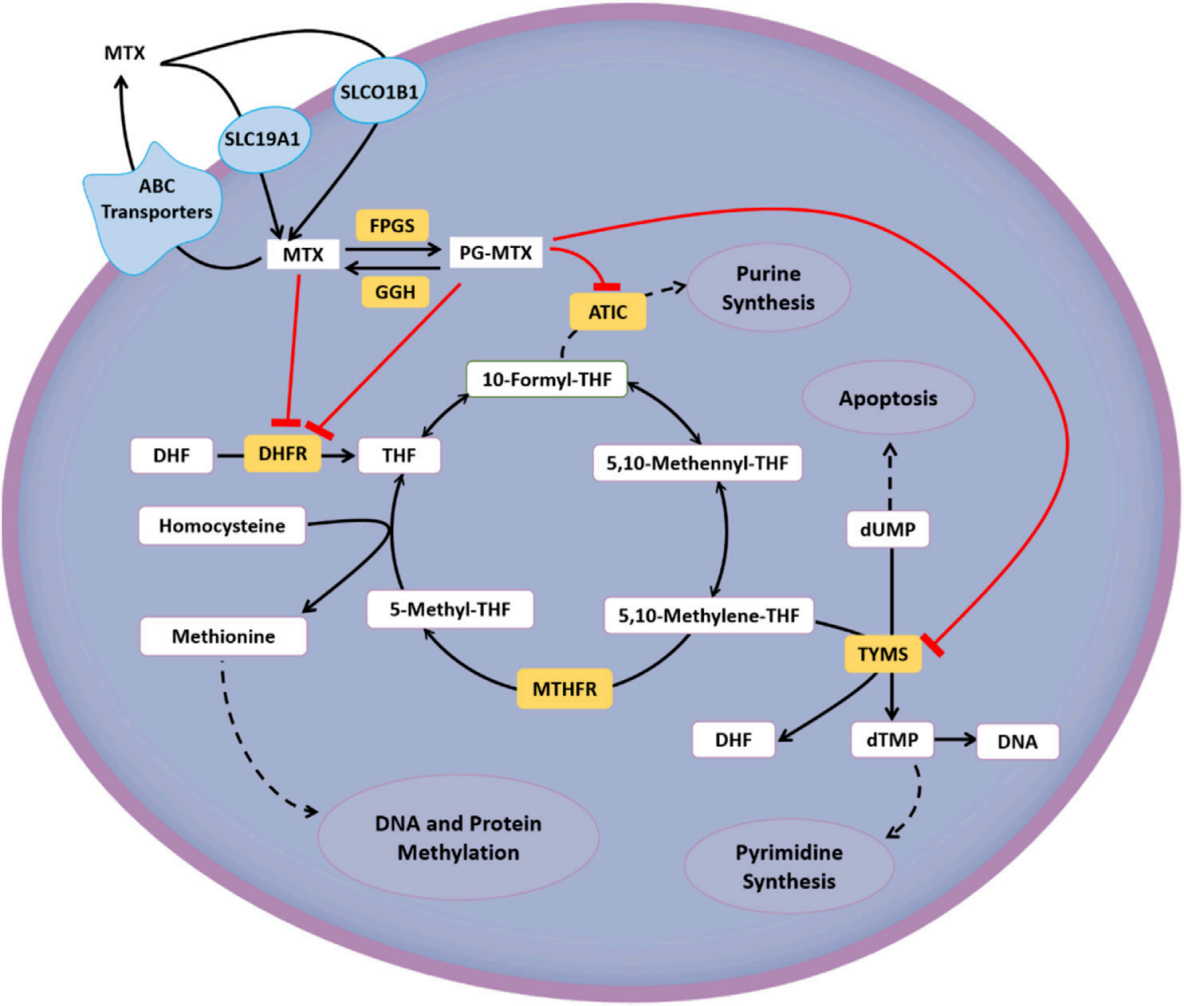
Cellular metabolic pathway and targets of HDMTX.

The interindividual diversity in the response to HDMTX can be partially explained by genetic variations involved in the MTX pathway, including cellular transport, drug metabolism, and target (Giletti and Esperon, 2018). Regarding transcellular transport, the cellular influx and efflux are mainly mediated by the reduced folate carrier 1 (RFC1/SLC19A1) (Zhao et al., 2011) and ATP-binding cassette transporters (ABC, predominantly ABCB1) (Assaraf, 2006), respectively. In some tissues, the influx process is related to organic anion transporting polypeptides (OATP/SLCO) (Wang et al., 2019). Regarding the polyglutamation pathway, MTX is converted into polyglutamate forms (PGMTX) by the enzyme folylpolyglutamate synthetase (FPGS) once inside the cell, and this process can be reversed by the enzyme gamma-glutamyl hydrolase (GGH) (Giletti and Esperon, 2018). Regarding the target, dihydrofolate reductase (DHFR), thymidylate synthase (TYMS/TS), and 5-aminoimidazole-4-carboxamide ribonucleotide transformylase (ATIC) are main therapeutic targets of HDMTX. And it has an indirect effect on methylenetetrahydrofolate reductase (MTHFR) (Giletti and Esperon, 2018). The cellular metabolic pathway and targets of HDMTX are summarized in Figure 1.

In recent years, pharmacogenetics of MTX has become a wide clinical concern and research focus. Numerous pharmacogenetic studies have evaluated the associations of HDMTX genetic polymorphism and outcomes (Avivi et al., 2014; Yang et al., 2017; Kotur et al., 2020), whereas the conflicting and contrasting evidence complicates the clinical utility. Six systematic reviews focusing on hematological malignancies have also been published and reported inconsistent findings (Yang et al., 2012; Lopez-Lopez et al., 2013; He et al., 2014; Zhao et al., 2016; Oosterom et al., 2018; Yao et al., 2019). However, most of systematic reviews did not set strict restrictions on the high dose (HDMTX) (Yang et al., 2012; Lopez-Lopez et al., 2013; He et al., 2014; Zhao et al., 2016; Yao et al., 2019), although the side effect profile of MTX varies markedly as its dose changes, and the pharmacogenetic associations may differ. Obviously, studies included in the latest systematic review (Yao et al., 2019) were published before January 2018, and the included data might be out of date. For example, four recent cohort studies (Chae et al., 2020; Esmaili et al., 2020; Kotur et al., 2020; Chang et al., 2021) investigating HDMTX pharmacogenetics were published in 2020. In addition, previous systematic reviews only included individual polymorphisms, focusing on toxicity but not prognosis outcomes (He et al., 2014; Zhao et al., 2016; Oosterom et al., 2018). Currently, there still exists gap between pharmacogenetic research and genetic testing in clinical practice. Predicting the toxic effects and tailoring HDMTX doses still remain an unmet clinical need in HDMTX therapy.

Thus, we conducted a systematic review to assess the association between gene polymorphisms within the HDMTX pathway and HDMTX toxicity or response in patients with hematological malignancies, aiming to provide applicable evidence for further personalized medications and fill the gap between knowledge and clinical practice.

## Methods

This study was performed according to the Preferred Reporting Items for Systematic Reviews and Meta-Analysis (PRISMA) statement (Moher et al., 2009). The PRISMA checklist was included in Supplementary Material I (Supplementary Table S1). The protocol for this systematic review has been registered in the International Prospective Register of Systematic Reviews (PROSPERO, No. CRD42018096986).

### Eligibility Criteria

Studies were considered eligible if they satisfied all of the following inclusion criteria: 1) type of studies: cohort study; 2) type of subject: patients with ALL, NHL, and other hematological malignancies receiving HDMTX chemotherapy, with no restrictions on ethnicity, gender, or age; 3) classification of exposure: patients were grouped by wild or mutant genotype of included genes within the HDMTX pathway (Table 1). There were 12 genetic polymorphisms in total, including but not limited to genes involved in the Pharmacogenomics Knowledge Base (PharmGKB, https://www.pharmgkb.org/guidelineAnnotations), which contains recommendations from the Clinical Pharmacogenetics Implementation Consortium (CPIC) and other national association of pharmacogenomics; and 4) types of outcomes measured: HDMTX-related toxicity and prognosis outcomes. The toxicity outcomes included the rate of hepatic toxicity, renal toxicity, oral mucositis, gastrointestinal (GI) toxicity, neurotoxicity, dermal toxicity, overall toxicity, and therapeutic interference due to toxicity. The toxicity outcomes were identified by the Common Terminology Criteria for Adverse Events established by American National Cancer Institute (NCI-CTC) or Toxicity Grading Scale for Determining the Severity of Adverse Events of Chemotherapeutic Drugs established by the World Health Organization (WHO) or other common criteria. Grade 3 to 4 (G3-4) indicates severe toxicity. The prognosis outcomes included overall survival (OS), progression-free survival (PFS), disease-free survival (DFS), event-free survival (EFS), relapse-free survival (RFS), and relapse/death. The exclusion criteria were as follows: duplicate publications; abstracts without available full texts; unqualified data; and studies not in accordance with the Hardy–Weinberg equilibrium (HWE) (Trikalinos et al., 2006) or not reporting the genotype distribution.

**TABLE 1:** Genetic polymorphisms within the HDMTX pathway

Gene	SNP	Polymorphisms	Remark
Transcellular transport			
*RFC1/SLC19A1*	rs1051266	80A>G	Research focus
*SLCO1B1*	rs4149056	521T>C	
*ABCB1*	rs1045642	3435C>T	Research focus
Polyglutamation pathway			
*FPGS*	rs10106	1994A>G	
*FPGS*	rs1544105	2752G>A	
*GGH*	rs3758149	401C>T	
Targets			
*MTHFR*	rs1801133	677C>T	Research focus
*MTHFR*	rs1801131	1298A>C	Research focus
*DHFR*	rs408626	317A>G	
*DHFR*	rs442767	680C>A	
*TYMS/TS*	rs34743033	2R/3R	
*ATIC*	rs2372536	347C>G	

### Search Strategy

Electronic databases including PubMed, Embase, Cochrane Central Register of Controlled Trials (CENTRAL), and Clinical Trials.gov were searched for potentially relevant studies from inception to November 11, 2020. Specific search strategies were developed for each database. The combination of keywords (“Methotrexate”) AND ("Hematologic neoplasms” OR Hematologic malignancy” OR “Leukemia” OR “Lymphoma”) AND (“Gene” OR “Polymorphism” OR “Pharmacogenetics” OR “Polymorphism, single nucleotide”) were used to search the title and abstract of queried literature (Supplementary Material II). No restrictions were placed on study design or language. The reference lists of previous systematic reviews and included literature were searched manually.

### Study Selection

Two authors (ZS and YH) independently assessed the eligibility of all studies based on the aforementioned inclusion and exclusion criteria after reviewing the study title, abstract, and full text in succession. Studies were included in only the systematic review (but not the meta-analysis) if their findings were relevant to the research question, but data were not available for quantitative analysis. Any disagreement among authors was discussed and reconciled by the corresponding author (RZ).

### Data Extraction

Two authors (ZS and YH) independently extracted data based on a predesigned standardized extraction form, including the first author and publication year, country, ethnicity, diagnosis, sample size and genotype distribution, gender (female/male), age (years), MTX dose, calculated *p*-value for HWE, outcomes, and individual results of the single study. Study authors were contacted for missing data.

### Quality Assessment/Risk of Bias

Two authors (ZS and YH) independently assessed the quality of studies under the Newcastle–Ottawa Scale (NOS) (Stang, 2010), as recommended in the Cochrane Handbook. The NOS attributes a maximum of 9 points to studies based on methodological design and formal reporting, involving “selection of cohorts,” “comparability of cohorts,” and “assessment of outcome.” NOS scores ranging from 7 to 9 points indicate high quality, 5 to 6 indicate medium quality, and 0 to 4 indicate low quality. Disagreements regarding data extraction and quality assessment were resolved by consensus or, when necessary, by consulting the corresponding author (RZ).

### Statistical Analyses

A chi-square test was performed to verify genotype distributions using SPSS version 25.0. A *p*-value greater than 0.05 would indicate accordance with the HWE. Before conducting the meta-analysis, clinical heterogeneity was estimated by comparing the diagnosis, efficacy or toxicity criteria, and other clinical features among studies. If two or more studies reported the same outcome and obvious clinical heterogeneity was not observed, meta-analysis was performed to quantitatively integrate outcomes by using the Cochrane Collaboration review manager software 5.3 (RevMan 5.3). Otherwise, only a descriptive analysis was performed.

The meta-analysis was performed as follows: 1) odds ratios (ORs) and hazard ratios (HRs) were calculated to evaluate the genetic association of toxicity or prognosis outcomes, respectively. And if the corresponding 95% confidence intervals (95% CIs) of the OR value (HR value) did not overlap with the value of 1 and the *p*-value was less than 0.05, the association was considered statistically significant. 2) The pooled OR or HR was calculated under the dominant model (MM/Mm vs mm), recessive model (MM vs Mm/mm), and allelic model (M vs m), where M is the mutant allele such that the G allele at *RFC1 80A>G*, C allele at *SLCO1B1 521T>C*, T allele at *ABCB1 3435C>T*, and so on; m is the wild allele, such that the A allele at *RFC1 80A>G*, T allele at *SLCO1B1 521T>C*, C allele at *ABCB1 3435C>T*, and so on. 3) The fixed-effect model was used initially, and the random-effects model was adopted when unidentified significant heterogeneity was detected. 4) The heterogeneity across the studies was assessed using a chi-square-based Q-test and I^2^ statistics. P_heterogeneity_ (P_het_) values <0.05 and I^2^ values >50% were considered to indicate significant heterogeneity (Sedgwick, 2015), and in these cases, the source of heterogeneity was investigated by examining the steps taken to check data and perform the subgroup analysis and sensitivity analysis. If the potential sources of heterogeneity remained unclear, the random-effects model was used, or the descriptive analysis was performed. 5) Subgroup analyses were performed based on patients’ age. Age subgroups were defined as pediatric, adult, and mixed-age. P_subgroup_ (P_sub_) <0.05 indicated a statistically significant difference across subgroups.

### Sensitivity Analyses and Publication Bias Assessment

Sensitivity analyses were conducted to assess the impact of individual studies on the pooled estimates and the stability of the pooled estimates. A pooled OR (HR) was recalculated after removing each single primary study one by one and replacing the statistical model of meta-analysis. Publication bias was assessed by inspecting the funnel plot visually, and it was considered to be valid when 10 or more studies were included (Sedgwick and Marston, 2015).

## Results

### Electronic Searches and Study Selection

**FIGURE 2:**
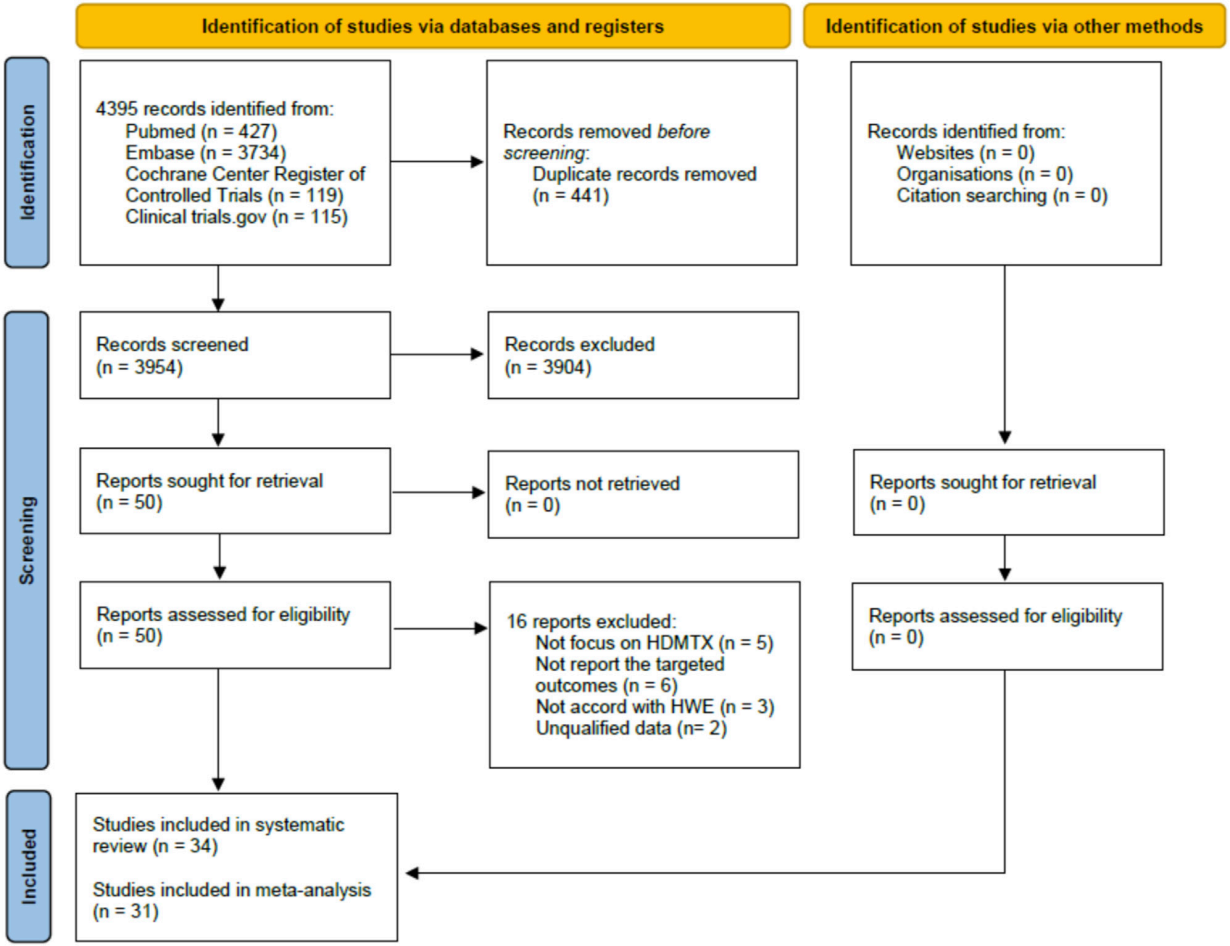
PRISMA 2020 flow diagram of studies selection for the systematic review and meta-analysis.

A total of 4395 candidate references were identified in electronic database searches, and no additional reference was identified using a manual search. Of the total 4395 candidates, 441 duplicate references were removed and then 3904 were excluded after careful review of the titles and abstracts. Only 50 references were recognized as relevant and then we assessed all full texts. The PRISMA 2020 flow diagram is shown in Figure 2. Of the total 50 references, five did not focus on HDMTX, six did not report the targeted outcomes, three did not accord with HWE, and two did not have qualified data. Finally, according to the aforementioned inclusion and exclusion criteria, 34 studies were included in our systematic review. Of the 34 studies (Laverdière et al., 2002; Kishi et al., 2003; Seidemann et al., 2006; Shimasaki et al., 2006; Imanishi et al., 2007; Ruiz-Argüelles et al., 2007; Ashton et al., 2009; Faganel Kotnik et al., 2010; D'Angelo et al., 2011; Faganel Kotnik et al., 2011; Liu et al., 2011; Chiusolo et al., 2012; Erčulj et al., 2012; Haase et al., 2012; Fukushima et al., 2013; Radtke et al., 2013; Yanagimachi et al., 2013; Avivi et al., 2014; Erculj et al., 2014; Suthandiram et al., 2014; den Hoed et al., 2015; Ma et al., 2015; Huang et al., 2016; Tsujimoto et al., 2016; Choi et al., 2017; Giletti et al., 2017; Liu et al., 2017; Yang et al., 2017; Yazıcıoğlu et al., 2017; Oosterom et al., 2018; Chae et al., 2020; Esmaili et al., 2020; Kotur et al., 2020; Chang et al., 2021) included, 31 studies were included in the meta-analysis and three studies were only included for the descriptive analysis since the meta-analysis was infeasible.

### Study Characteristics and Quality Assessment

In total, 34 studies involving 4102 patients were included for investigating the associations between genetic polymorphisms and HDMTX outcomes. Of the studies included, 14 studies reported the polymorphisms of *RFC1 (rs1051266)*, six studies *SLCO1B1 (rs4149056*), seven studies *ABCB1 (rs1045642)*, one study *FPGS (rs10106)*, one study *FPGS (rs1544105)*, 26 studies *MTHFR (rs1801133)*, 17 studies *MTHFR (rs1801131)*, six studies *TYMS (rs34743033)*, and one study *ATIC (rs2372536)*. None of studies reported the polymorphisms of *GGH (rs3758149)* and *DHFR (rs408626*, *rs442767)* yet*.* Among included studies, 20 and 14 studies were conducted in the ethnicity of Caucasian and Asian, respectively. Patients’ diagnosis included ALL, acute myeloid leukemia (AML), diffuse large B-cell lymphoma (DLBCL), primary CNS lymphoma (PCNSL), and other NHL. A total of 25 studies included pediatric patients only, 8 studies included adult patients only, and one study did not impose restrictions on patient age. All the studies were in accord with the HWE. And 30 studies reported toxicities outcomes, while prognosis outcomes were involved in 14 studies. Regarding quality assessment, 2 studies earned a full NOS score of 9 points, 19 studies earned 8 points, and 11 studies earned 7 points, varying mainly in presentation of the outcomes at the start of study and outcome follow-up. The average NOS score of all studies was 7.6 points, indicating a relatively high quality of overall methodology. The main characteristics and NOS scores of the studies included are summarized in Table 2. And the detailed NOS scores of the studies included are given in Supplemental Material III (Supplementary Table S2).

**TABLE 2:** Main characteristics and NOS scores of the studies included

Author-year	Country	Ethnicity	No. of cases	F/M	Age (y)	HWE	Outcome	*RFC1/SLC19A1*	*SLCO1B1*	*ABCB1*	*FPGS*	*MTHFR*	*MTHFR*	*TYMS/MS*	*ATIC*	NOS score
								*80 A>G rs1051266*	*521 T>C rs4149056*	*3435 C>T rs1045642*	*2752 G > A*, *rs1544105; 1994 A>G*, *rs10106*	*677 C>T rs1801133*	*1298 A>C rs1801131*	*2R>3R rs34743033*	*347 C>G rs2372536*	
Esmaili M A-2020	Iran	Caucasian	74	28/46	Median 5, pediatric	Yes	Tox^a^, Prog^b^	AG↗ Tox		T↘ Prog		TT↘ Prog	AC↘ Tox			7
Kotur N-2020	Serbia	Caucasian	148	54/94	Median 5.5 (0.9–17.6)	Yes	Tox	G↘ Tox	NS^c^			NS	NS			7
Liu S G-2017	China	Asian	322	NR	Median 4 (1.0–15.0)	Yes	Tox, Prog	NS	CC↘ Prog	NS						7
Den Hoed M-2014	Netherlands	Caucasian	134	64/70	Median 5.3 (1.4–18.1)	Yes	Tox	NS	NS			NS	NS			8
Suthandiram S-2014	Malaysia	Asian	71	35/36	36.6 ± 14.2	Yes	Tox	G↘ Tox		T↗ Tox		TT↗ Tox	NS			8
Yanagimachi M-2013	Japan	Asian	51	25/26	Median 5.9 (1–15)	Yes	Tox	NS				NS	NS			6
Chiusolo P-2012	Italy	Caucasian	54	25/29	Median 52 (15–78)	Yes	Tox, Prog	GG↘ Prog				NS	C↘ Tox			8
Faganel K B-2011	Slovenia	Caucasian	64	38/26	Median 5 (1.6–16.8)	Yes	Tox	NS		NS		T↗ Tox	NS	3R↘ Tox		8
Faganel K B-2010	Slovenia	Caucasian	60	37/23	Pediatric	Yes	Tox, Prog	NS								8
Ashton L J-2009	Australia	Caucasian	170	NR	Pediatric	Yes	Prog	GG↘ Prog				NS				8
Imanishi H-2007	Japan	Asian	26	10/16	6.7 ± 4.7	Yes	Tox	NS				NS				8
Shimasaki N-2006	Japan	Asian	15	9/6	Median 6 (1–14)	Yes	Tox	G↗ Tox				NS				8
Kishi S-2003	America	Caucasian	53	23/30	Median 6 (0–18)	Yes	Tox	NS				NS				7
Laverdiere C-2002	Canada	Caucasian	204	92/112	Pediatric	Yes	Tox, Prog	GG↘ Tox, GG↗ Prog								7
Yang L-2017	China	Asian	105	33/72	42.5 ± 17.9	Yes	Tox		C↗ Tox		1994 A>G NS					8
Avivi I-2014	Israel	Caucasian	69	20/49	Median 56 (25–83)	Yes	Tox		C↘ Tox	NS		NS	NS			9
Fukushima H-2013	Japan	Asian	103	41/62	Median 7.43 (0.2–19.2)	Yes	Tox, Prog		NS			T↗ Tox	C↗ Prog, C↗ Tox			8
Tsujimoto S-2016	Japan	Asian	56	27/29	Median 5 (0–15)	Yes	Tox			NS		NS			NS	8
Ma C X-2015	China	Asian	178	72/106	Median 30 (18–59)	Yes	Tox, Prog			T↗ Tox						8
Chae H-2020	Korea	Asian	117	35/82	Median 9 (5–13)	Yes	Tox					TT↗Tox	NS			8
Chang X-2021	China	Asian	32	11/21	≥14	Yes	Tox					TT↗Tox				8
Giletti-2017	Uruguayan	Caucasian	41	12/29	36 ± 13.9	Yes	Tox					NS				7
Yazicioglu B-2017	Turkey	Caucasian	106	39/67	Median 5 (1–17)	Yes	Tox, Prog					NS	NS	NS		7
Choi Y J-2016	Korea	Asian	111	53/58	Median 60 (17–86)	Yes	Tox					T↗Tox				8
Erculj N-2014	Slovenia	Caucasian	29	4/25	Median 11 (1–8)	Yes	Tox					T↗Tox	NS	NS		8
Erculj, N-2012	Slovenia	Caucasian	167	87/80	Median 4.7 (0.3–18)	Yes	Tox, Prog					NS	NS	NS		8
Haase R-2012	Germany	Caucasian	34	17/17	7.1 ± 4.8	Yes	Tox					NS	NS			7
D'Angelo V-2011	Italy	Caucasian	151	48/103	Pediatric	Yes	Prog					TT↘Prog	NS			7
Liu S G-2011	China	Asian	181	66/115	5.7 ± 3.6	Yes	Tox					NS	C↘Tox			8
Ruiz-Arg elles G J-2007	Mexico	Caucasian	28	7/21	Mean 16.5 (0–40)	Yes	Tox					NS				6
Seidemann-2006	Austria, Germany, Switzerland	Caucasian	484	144/340	Pediatric	Yes	Prog					NS				7
Huang Z-2016	China	Asian	57	26/31	5.9 ± 4.3	Yes	Prog				2752 AA↗Prog					9
Oosterom N-2017	Netherlands	Caucasian	108	49/59	Median 5.7 (1–18)	Yes	Tox							NS		7
Radkte S-2013	Germany	Caucasian	499	204/295	6.4 ± 4.0	Yes	Tox, Prog						CC↘Prog	3R↘Tox		8

Note:↗: increase; ↘: reduce.

Abbreviation:

a Tox: toxicity.

b Prog: prognosis.

c NS: no significant association between the genetic polymorphisms and the outcomes.

### Overall Findings

The overall findings are summarized in Table 3. Regarding the cellular transport and metabolism, *RFC1 (rs1051266)* was associated with a reduced risk of hepatic toxicity and overall toxicity (in pediatric), while *ABCB1 (rs1045642)* was associated with an increased risk of hepatic toxicity. No association was observed in other toxicities outcomes and genetic polymorphisms. According to findings of prognostic outcomes from individual studies, *RFC1 (rs1051266)* was associated with worse 2y-OS and 2y-PFS (in adult), and *SLCO1B1 (rs4149056)* was associated with worse 5y-EFS (in pediatric). However, *FPGS (rs1544105)* was associated with better 2y-OS (in adult). No association was observed in other outcomes of relapse.

**TABLE 3:** Overall findings

	Dominant model	Recessive model	Allelic model	Other findings of prognosis^a^
***RFC1* A80G (rs1051266)**	GG/AG vs AA	GG vs AG/AA	G vs A	
Hepatotoxicity	**=**	**↘**	**=**	
G3-4 hepatotoxicity (pediatric)	**=**	No data	No data	
Renal toxicity	**=**	**=**	**=**	
Mucositis	**=**	**=**	**=**	
G3-4 mucositis (pediatric)	**=**	**=**	**=**	
Neurotoxicity (pediatric)	**=**	**=**	**=**	
G3-4 neurotoxicity (pediatric)	**=**	**=**	**=**	
GI^b^ toxicity (pediatric)	**=**	No data	**=**	
Overall toxicity (pediatric)	**=**	**↘**	**=**	
Therapeutic interference (pediatric)	**=**	**=**	**=**	
Relapse (pediatric)	**=**	**=**	**=**	
2y-PFS (adult)	No data	**↘**	No data	NR^c^
2y-OS (adult)	No data	**↘**	No data	NR
5y-EFS (pediatric)	No data	**=**	No data	NR
***SLCO1B1* T521C (rs4149056)**	**CC/TC vs TT**	**CC vs TC/TT**	**C vs T**	
Hepatotoxicity	**=**	No data	No data	
Renal toxicity (pediatric)	**=**	No data	No data	
G3-4 mucositis (pediatric)	**=**	**=**	**=**	
GI toxicity (pediatric)	No data	**=**	No data	
Overall toxicity (adult)	**=**	**=**	**=**	
Relapse (pediatric)	**=**	No data	No data	
5y-EFS (pediatric)	No data	**↘**	No data	NR
***ABCB1* C3435T (rs1045642)**	**TT/CT vs CC**	**TT vs CT/CC**	**T vs C**	
Hepatotoxicity	**↗**	**=**	**=**	
Renal toxicity (adult)	No data	**=**	No data	
Mucositis	**=**	**=**	**=**	
G3-4 Mucositis (pediatric)	**=**	**=**	**=**	
Neurotoxicity (pediatric)	**=**	**=**	**=**	
GI toxicity (adult)	**=**	No data	No data	
Overall toxicity (adult)	**=**	**=**	**=**	
Therapeutic interference (pediatric)	**=**	**=**	**=**	
EFS	**= or↘**	No data	No data	NR
***FPGS* A1994G (rs10106)**	**GG/AG vs AA**	**GG vs AG/AA**	**G vs A**	
Hepatotoxicity (adult)	No data	**=**	No data	
***FPGS* G2752A (rs1544105)**	**AA/GA vs GG**	**AA vs GA/AA**	**A vs G**	
2y-OS (adult)	No data	**↗**	No data	NR
***MTHFR* C677T (rs1801133)**	**TT/CT vs CC**	**TT vs CT/CC**	**T vs C**	
Hepatotoxicity	**↗**	**↗**	**↗**	
G3-4 hepatotoxicity	**↘**	**=**	**=**	
Renal toxicity	**=**	**↗**	**↗**	
G3-4 renal toxicity (pediatric)	**=**	No data	No data	
Mucositis	**↗**	**=**	**↗**	
G3-4 mucositis (pediatric)	**=**	**=**	**=**	
G3-4 GI toxicity (pediatric)	**=**	**=**	**=**	
Dermal toxicity (pediatric)	**=**	**=**	**=**	
Neurotoxicity (pediatric)	**=**	**=**	**=**	
Overall toxicity (adult)	**=**	**=**	**=**	
Therapeutic interference (pediatric)	**↗**	**=**	**=**	
Relapse/death (pediatric)	**=**	**↗**	**=**	
5y-EFS (pediatric)	**=**	**=**	No data	**(TT vs CT vs CC) =**
RFS (pediatric)	**=**	No data	No data	NR
OS (pediatric)	**=**	No data	No data	NR
***MTHFR* A1298C (rs1801131)**	**CC/AC vs AA**	**CC vs AC/AA**	**C vs A**	
Hepatotoxicity	**=**	**=**	**=**	
G3-4 Hepatotoxicity (Pediatric)	**=**	**=**	**=**	
Renal toxicity	**=**	**=**	**↘**	
G3-4 renal toxicity (pediatric)	**=**	No data	No data	
Mucositis	**=**	**=**	**=**	
G3-4 mucositis (pediatric)	**=**	**=**	**=**	
GI toxicity (pediatric)	**=**	**=**	**=**	
G3-4 GI toxicity (pediatric)	**=**	**=**	**=**	
Dermal toxicity (pediatric)	**=**	**=**	**=**	
Neurotoxicity (pediatric)	**=**	**=**	**=**	
Overall toxicity (adult)	**=**	**=**	**=**	
Therapeutic interference (pediatric)	**=**	**=**	**=**	
Relapse/death (pediatric)	**=**	**=**	**=**	
EFS (pediatric)	= or**↗**	No data	No data	**(AC vs CC vs AA) =**
				**(CC vs AA) ↘**
RFS (pediatric)	**=**	No data	No data	NR
OS (pediatric)	**=**	No data	No data	NR
***TYMS/MS* 2R>3R (rs34743033)**	**3R3R/2R3R vs 2R2R**	**3R3R vs 2R3R/2R2R**	**3R vs 2R**	
Hepatotoxicity (pediatric)	**=**	No data	No data	
Renal toxicity (pediatric)	**=**	No data	No data	
Mucositis (pediatric)	**↘**	**=**	**=**	
G3-4 mucositis (pediatric)	**=**	**=**	**=**	
Neurotoxicity (pediatric)	**=**	**=**	**=**	
Overall toxicity (pediatric)	**=**	No data	No data	
RFS (pediatric)	**=**	No data	No data	NR
OS (pediatric)	**=**	No data	No data	NR
EFS (pediatric)	**=**	No data	No data	**(2R3R vs 3R3R vs 2R2R) =**
***ATIC* 347C>G (rs2372536)**	**GG/CG vs CC**	**GG vs CG/CC**	**G vs C**	
Neurotoxicity (pediatric)	**=**	**=**	No data	

**Note:** ↗: increase; ↘: reduce; =: no association between the genetic polymorphism and the outcome; = or↗: no association or increase; = or↘: no association or reduce.

a Several included studies reported outcomes of prognosis in other genetic models, and we also included these results for narrative analysis in the present review.

b GI: gastrointestinal.

c NR: not reported.

Regarding the target, the polymorphisms of *MTHFR* were the most extensively investigated genes. *MTHFR (rs1801133)* was associated with an increased risk of hepatic toxicity, renal toxicity, mucositis, and therapeutic interference (in pediatric). In contrast, *MTHFR (rs1801133)*, *MTHFR (rs1801131)*, and *TYMS (rs34743033)* were associated with a reduced risk of G3-4 hepatic toxicity, renal toxicity, and mucositis, respectively. Only one study investigated the polymorphisms of *ATIC (rs2372536)*, and it reported the lack of association of the neurotoxicity. According to findings of prognostic outcomes from individual studies, no association was observed.

### Meta-Analysis of Genetic Polymorphisms Within the Cellular Transport and Metabolism

#### The Association Between *RFC1* (rs1051266) and Toxicities and Prognosis Outcomes

The pooled OR (HR) of the associations for each outcome under three genetic models is summarized in Figure 3 and Supplementary Table S3. Regarding toxicity outcomes, significant associations were found in the outcomes of hepatotoxicity and overall toxicity. The pooled OR (95% CI) of hepatotoxicity was dominant, 0.62 (0.33–1.16); recessive, 0.35 (0.16–0.76) (Supplementary Figure S1); and allelic, 0.77 (0.48–1.21). The pooled OR of renal toxicity under three genetic models was 0.93 (0.41–2.10), 0.73 (0.21–2.56), and 0.91 (0.49–1.70), respectively. The pooled OR (95% CI) of mucositis under three genetic models was 0.91 (0.54–1.52), 0.99 (0.60–1.61), and 0.90 (0.61–1.32), respectively. Besides, a single study of pediatric patients (Laverdière et al., 2002) reported *RFC1* was associated with a reduced risk of overall toxicity (GG vs GA/AA: *p* < 0.05). Neither significant heterogeneity nor significant subgroup difference was detected in most comparisons. A moderate heterogeneity was only found in hepatotoxicity under the dominant model (P_het_ = 0.02, I^2^ = 65%). Regarding prognosis outcomes, qualitative analysis was performed since meta-analysis was unfeasible. Significant associations were found between *RFC1* and worse 2y-OS and 2y-PFS (GG vs GA/AA: *p* < 0.05), but the association was not observed in the outcome of relapse.

**FIGURE 3A-B:**
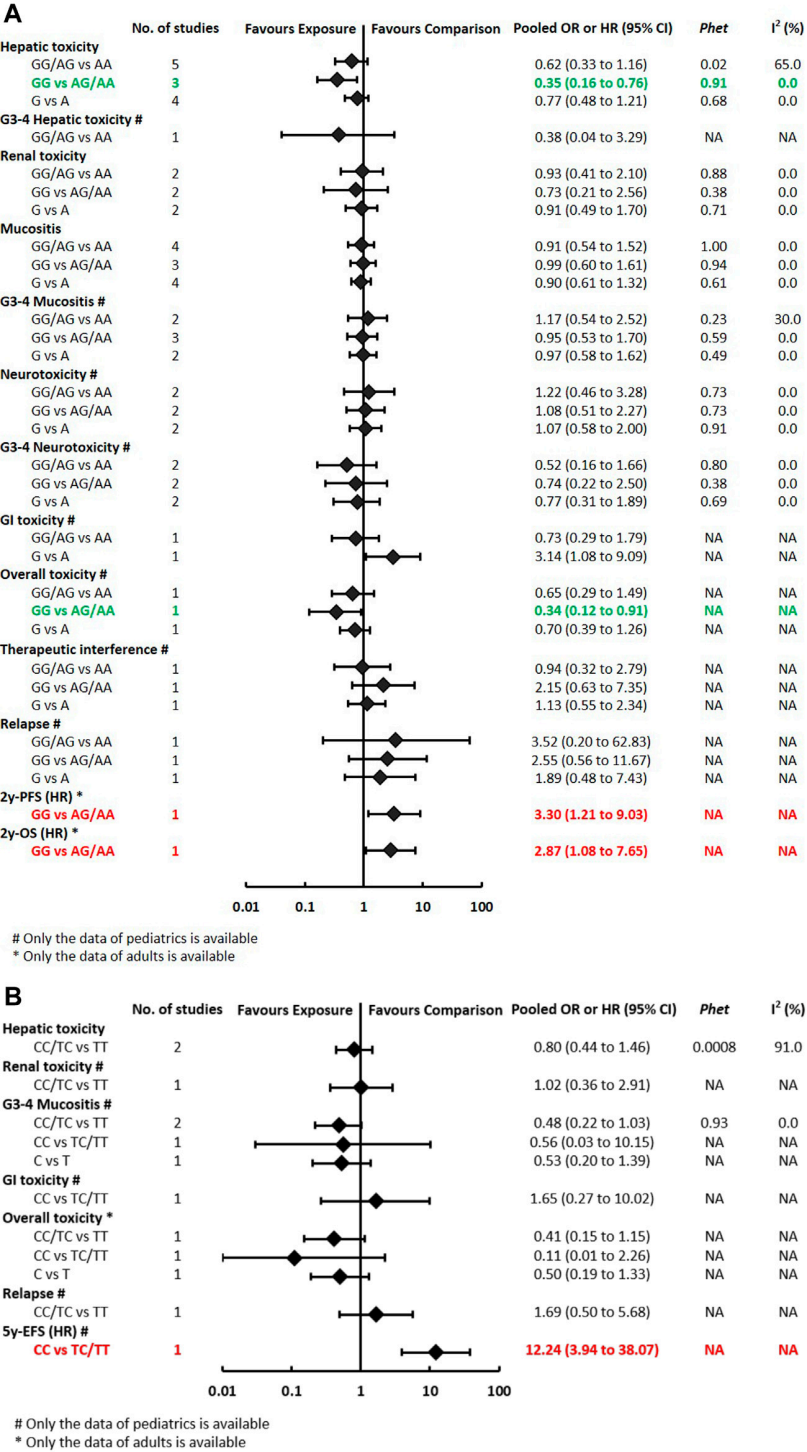
**(A)** Findings of the association between *RFC1 (rs1051266)* and HDMTX-related outcomes under three genetic models. **(B)** Findings of the association between *SLCO1B1 (rs4149056)* and HDMTX-related outcomes under three genetic models.

**FIGURE 3C-D:**
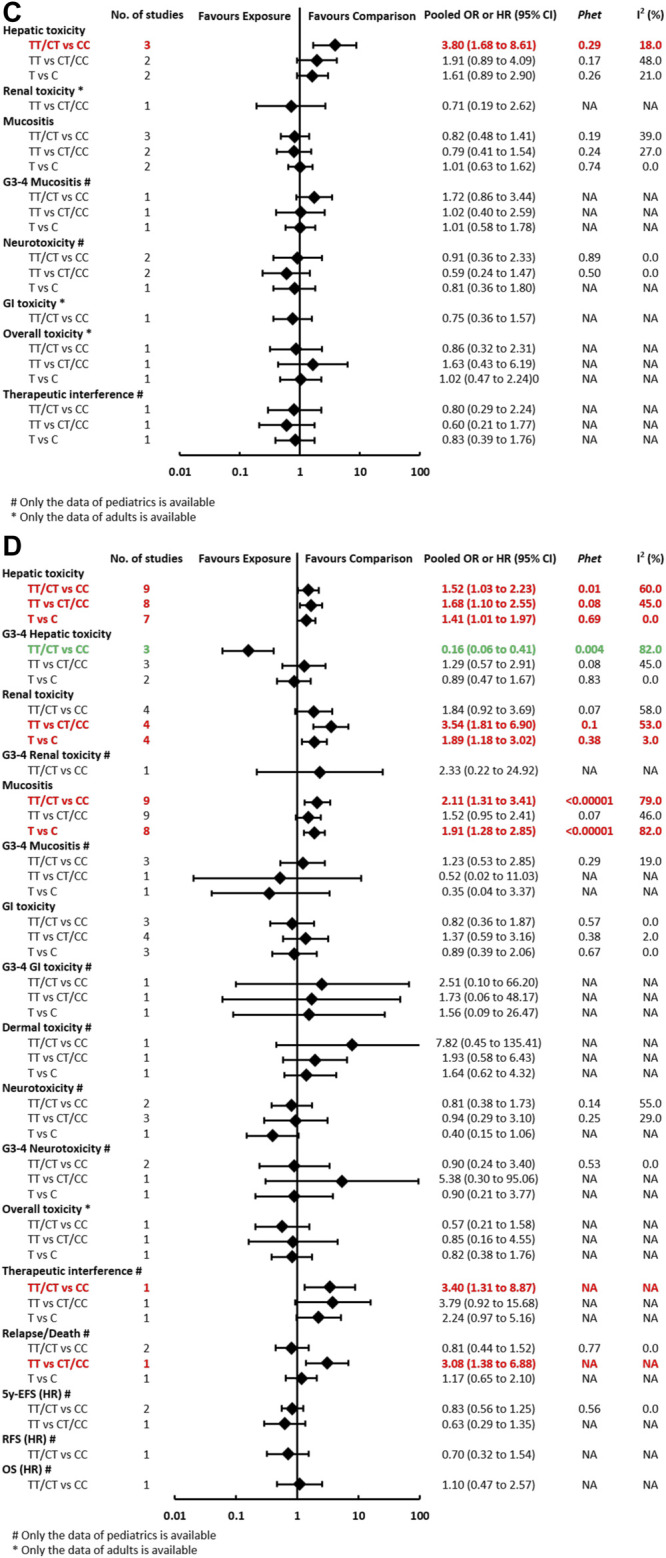
**(C)** Findings of the association between *ABCB1 (rs1045642)* and HDMTX-related outcomes under three genetic models. **(D)** Findings of the association between *MTHFR (rs1801133)* and HDMTX-related outcomes under three genetic models.

**FIGURE 3E-F:**
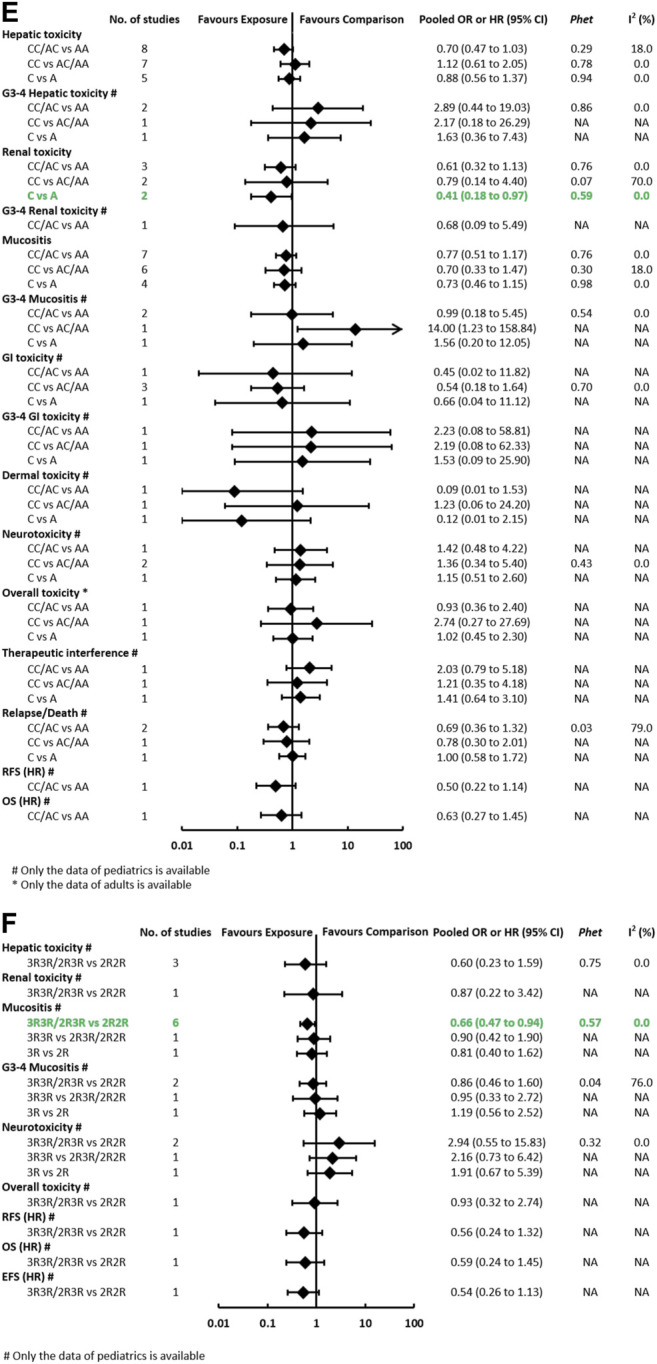
**(E)** Findings of the association between *MTHFR (rs1801131)* and HDMTX-related outcomes under three genetic models. **(F)** Findings of the association between *TYMS (rs34743033)* and HDMTX-related outcomes under three genetic models.

#### The Association Between *SLCO1B1* (rs4149056) and Toxicities and Prognosis Outcomes

The pooled OR (HR) of the associations for each outcome under three genetic models is summarized in Figure 3 and Supplementary Table S4. Regarding toxicity outcomes, no significant association was found in the outcomes of hepatic toxicity, renal toxicity, mucositis, and other toxicities. A considerable heterogeneity was detected in hepatotoxicity under the dominant model (P_het_ = 0.0008, I^2^ = 91%), which was partially related to significant differences among pediatric and adult subgroups (Supplementary Table S4). The pooled OR (95% CI) of pediatric and adult patients was 0.31 (0.13–0.76) and 3.05 (1.12–8.32), respectively. Regarding prognosis outcomes, qualitative analysis was performed since meta-analysis was unfeasible. Significant associations were found between *SLCO1B1 (rs4149056)* and worse 5y-EFS (CC vs TC/TT: *p* < 0.05), but the association was not observed in the outcomes of relapse.

#### The Association Between *ABCB1* (rs1045642) and Toxicities and Prognosis Outcomes

The pooled OR (HR) of the associations for *each outcome* under three genetic models is summarized in Figure 3 and Supplementary Table S5. Regarding toxicity outcomes, the pooled OR (95% CI) of hepatotoxicity was dominant, 3.80 (1.68–8.61) (Supplementary Figure S2); recessive, 1.91 (0.89–4.09); and allelic, 1.61 (0.89–2.90). Remarkably, for adult patients, the pooled OR (95% CI) of hepatotoxicity under the recessive model was 3.38 (1.07–10.68), which was inconsistent with the overall results of general population. However, neither significant heterogeneity nor significant subgroup difference was detected in all meta-analyses. Regarding prognosis outcomes, two studies (Ma et al., 2015; Esmaili et al., 2020) with conflicting results reported the outcome of EFS, so the association still remained ambiguous.

#### The Association Between *FPGS* (rs10106, rs1544105) and Toxicities and Prognosis Outcomes

The pooled OR (HR) of the associations is summarized at Supplementary Tables S6, S7. One study (Yang et al., 2017) reported no association of *FPGS (rs10106)* and the risk of hepatic toxicity in adults (GG vs AG/AA: OR = 0.60, 95% CI = 0.27–1.32). Conversely, another study (Huang et al., 2016) reported the association of *FPGS (rs1544105)* and better 2y-OS in adults, with the HR (95% CI) = 0.45 (0.24–0.84) under the recessive model.

### Meta-Analysis of Genetic Polymorphisms Within the Drug Targets

#### The Association Between *MTHFR* (rs1801133) and Toxicities and Prognosis Outcomes

The pooled OR (HR) of the associations for each outcome under three genetic models is summarized at Figure 3 and Supplementary Table S8. Regarding toxicity outcomes, significant associations were found in the outcomes of hepatotoxicity, G3-4 hepatotoxicity, renal toxicity, and mucositis. The pooled OR (95% CI) of hepatotoxicity was dominant, 1.52 (1.03–2.23) (Supplementary Figure S3); recessive, 1.68 (1.10–2.55); and allelic, 1.41 (1.01–1.97). On the contrary, the pooled OR (95% CI) of G3-4 hepatotoxicity under the dominant model was 0.16 (0.06–0.41). The pooled OR of renal toxicity under three genetic models was 1.84 (0.92–3.69), 3.54 (1.81–6.90), and 1.89 (1.18–3.02), respectively. The pooled OR (95% CI) of mucositis under three genetic models was 2.11 (1.31–3.41), 1.52 (0.95–2.41), and 1.91 (1.28–2.85), respectively. Significant heterogeneity was detected in several positive results, which can be explained partially by significant differences between pediatric and adult subgroups. Remarkably, the associations of hepatic toxicity or renal toxicity were observed in adults but not pediatric patients. However, the associations of mucositis were observed in pediatric but not adult patients (Supplementary Table S8). Regarding prognosis outcomes, significant association was found between *MTHFR (rs1801133)* and an increased risk of relapse/death (TT vs CT/CC: *p* < 0.05) in pediatric patients in a single study (D'Angelo et al., 2011), but the association was not observed in other outcomes of OS, RFS, and 5y-EFS.

#### The Association Between *MTHFR* (rs1801131) and Toxicities and Prognosis Outcomes

The pooled OR (HR) of the associations for each outcome under three genetic models is summarized at Figure 3 and Supplementary Table S9. Regarding toxicity outcomes, significant association was only found in the outcome of renal toxicity. The pooled OR (95% CI) of hepatotoxicity under three genetic models was 0.70 (0.47–1.03), 1.12 (0.61–2.05), and 0.88 (0.56–1.37), respectively. Notably, the association of hepatic toxicity was observed in pediatric (AC/CC vs AA: OR=0.59, 95% CI=0.37–0.92) but not adult patients. The pooled OR of renal toxicity under three genetic models was 0.61 (0.32–1.13), 0.79 (0.14–4.40), and 0.41 (0.18–0.97), respectively. The pooled OR (95% CI) of mucositis under three genetic models was 0.77 (0.51–1.17), 0.70 (0.33–1.47), and 0.73 (0.46–1.15), respectively. Neither significant heterogeneity nor significant subgroup difference was detected in all comparisons (Supplementary Table S9). Regarding prognosis outcomes in pediatric patients, two studies (Erčulj et al., 2012; Fukushima et al., 2013) with conflicting results reported the outcome of EFS, so the association still remained ambiguous. No association was observed in other prognosis outcomes of relapse/death, OS, and RFS.

#### The Association Between *TYMS* (rs34743033) and Toxicities and Prognosis Outcomes

The pooled OR (HR) of the associations for each outcome under three genetic models is summarized at Figure 3 and Supplementary Table S10. A meta-analysis of four studies showed that *TYMS (rs34743033)* was marginally associated with a reduced risk of mucositis under the dominant model (OR = 0.66, 95% CI = 0.47–0.94) (Supplementary Figure S4). No association was observed in the outcomes of hepatotoxicity, renal toxicity, neurotoxicity, and overall toxicity. Regarding prognosis outcomes, meta-analysis was unfeasible since only single study (Erčulj et al., 2012) reported the same outcomes, and no association was found in outcomes of OS, EFS, and RFS.

#### The Association Between *ATIC* (rs2372536) and Toxicities

The pooled OR of the associations is summarized at Supplementary Table S11. Only one study (Tsujimoto et al., 2016) investigated the relationship between *ATIC (rs2372536)* and neurotoxicity in pediatric patients and did not report the presence of an association.

### Sensitivity Analyses

**FIGURE 4:**
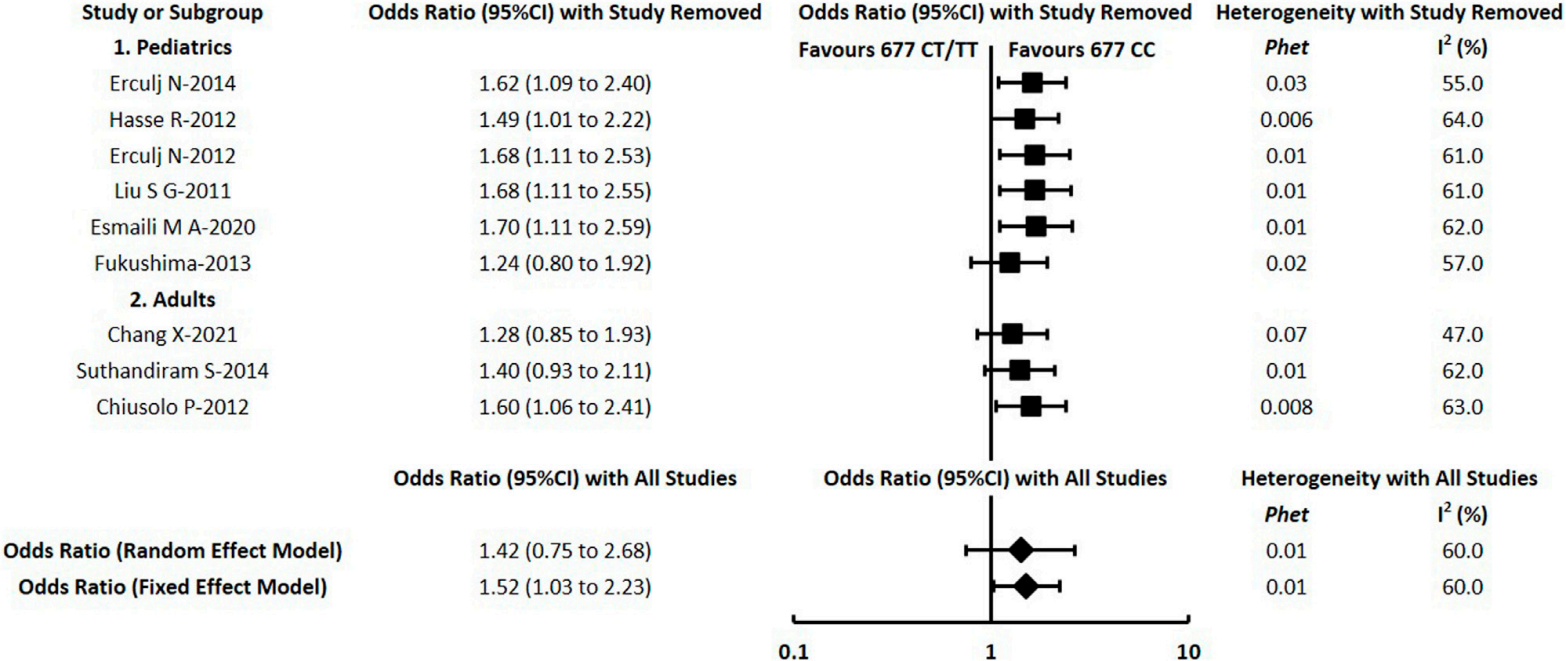
Sensitivity analysis for meta-analysis of the association between *MTHFR (rs1801133)* and hepatotoxicity under the dominant model.

To assess the impact of individual studies on the overall pooled estimate and explore potential sources of heterogeneity, sensitivity analyses were conducted by removing each study one by one for each comparison. In a total of 66 meta-analyses in this study, substantial changes were indicated in a small proportion of comparisons. For the meta-analysis of *RFC1 (rs1051266)*, the outcome of hepatotoxicity under the dominant model changed to OR = 0.39 with 95% CI = 0.19–0.80 after excluding Esmali 2020 (Esmaili et al., 2020). For the *MTHFR (rs1801133)*, the statistically significant result of hepatotoxicity under the dominant model changed substantially after excluding Chang 2021 (Chang et al., 2021) or Suthandiram 2014 (Suthandiram et al., 2014) or Fukushima 2013 (Fukushima et al., 2013) or switching into the random-effects model (Figure 4). Similarly, the substantial changes of pooled OR (95% CI) were detected in the following comparisons of *MTHFR (rs1801133)*: the result of hepatotoxicity under recessive and allelic models after excluding Chang 2021 (Chang et al., 2021) or Suthandiram 2014 (Suthandiram et al., 2014); the renal toxicity under recessive and allelic models after excluding Chang 2021 (Chang et al., 2021); the mucositis under dominant and allelic models after excluding Faganel 2011 (Faganel Kotnik et al., 2011); and the mucositis under the recessive model after excluding Suthandiram 2014 (Suthandiram et al., 2014). However, the pooled estimate of all the other comparisons did not change significantly when different data were used, indicating that the conclusions of this study had a certain degree of reliability.

### Publication Bias Assessment

Publication bias was evaluated by analyzing the funnel plots visually. Serious publication bias was not indicated in any of the outcomes. For instance, the funnel plot (Supplementary Figure S5) does not suggest any serious bias for hepatotoxicity under the dominant model of *MTHFR (rs1801133)*. However, it is notable that publication bias for some outcomes could not be excluded entirely by visual inspection of the funnel plots.

## Discussion

### General Findings and Trends

Recently, investigations into the HDMTX response from the perspective of genetic variation have been relatively recent and wide in scope. In this study, we conducted a systematic review aiming to identify and summarize present evidence evaluating the associations between genetic polymorphisms with HDMTX toxicity and prognosis outcomes. With the aim of identifying relevant variants that could be implemented in clinical prediction, we reviewed and investigated genetic polymorphisms within the whole pathway of drug metabolism and targets in this study. As HDMTX is far more toxic than low-dose MTX (Schmiegelow, 2009), patients with malignant cancer receiving HDMTX are more easily associated with serious toxic responses than those with rheumatoid arthritis. Besides, the dosage and infusion regimens of HDMTX vary greatly in the treatment of hematological malignancies and osteosarcoma (Ramsey et al., 2018), so toxic responses can be markedly different in the two malignant cancers. Therefore, we pay more attention to patients with hematological malignancies in this present study.

The polymorphisms of *RFC1 (rs1051266)*, *ABCB1 (rs1045642)*, and *MTHFR (rs1801133*, *rs1801131)* were the research focus in current pharmacogenetic studies of HDMTX. The most investigated clinical outcomes were hepatic toxicity and mucositis, followed by renal toxicity, while prognostic outcomes were reported by a small proportion of studies (14/34). Generally speaking, our study confirmed that the *MTHFR 677C>T (rs1801133)* has a significant effect on the increased risk of HDMTX toxicities (including hepatotoxicity, mucositis, and renal toxicity), and the *ABCB1 3435C>T (rs1045642)* has a significant effect on the increased risk of hepatotoxicity, which corresponds to the findings in previous studies (Yang et al., 2012; Zhao et al., 2016; Zhu et al., 2018; Yao et al., 2019; Maagdenberg et al., 2021), whereas we found a tendency toward reduced risk of hepatotoxicity in carriers of *RFC1 80GG* and toward reduced risk of mucositis in those with *TYMS 3R3R or 2R3R* genotypes. Also, a tendency toward reduced risk of renal toxicity was observed in carriers with the *MTHFR 1298 variant C allele*, which was similar to the results of mucositis and GI toxicity (Zhao et al., 2016) and dermal toxicity (Yang et al., 2012). In other words, a protective effect of some genetic polymorphisms on developing individual toxicities is suggestive in our study. It is worth mentioning that Lopez-Lopez 2013 (Lopez-Lopez et al., 2013) reported no association between *MTHFR 677C>T* and MTX toxicity in pediatric ALL (recessive model), and Oosterom 2018 (Oosterom et al., 2018) reported no association between *TYMS 2R>3R* and MTX toxicity in pediatric ALL (dominant model), which were contrary to our findings. The inconsistent patient’s age and MTX dosage may explain the discrepancy in different findings. With regard to descriptive analysis, limited evidence from single studies showed significant associations in the following prognosis outcomes: *RFC1 (rs1051266)* and worse 2y-PFS and 2y-OS; *SLCO1B1 (rs4149056)* and worse 5y-EFS; and *FPGS (rs1544105)* and better 2y-OS. Besides, a lack of association was observed in other outcomes investigated in our review.

Since HDMTX-related tolerance and toxicities might be influenced by patients’ age in the real clinical practice (Zhu et al., 2018), subgroup analysis was conducted to explore the difference. Significant differences were suggestive in the following outcomes: hepatotoxicity (*SLCO1B1 rs4149056*; *MTHFR rs1801133)*, renal toxicity (*MTHFR rs1801133)*, and mucositis (*MTHFR rs1801133)*. It is worth mentioning that in the meta-analysis of *MTHFR (rs1801133)*, the aforementioned subgroup differences can explain the heterogeneity of those outcomes with positive findings (hepatotoxicity, renal toxicity, and mucositis) to a certain extent. And patients’ age is possibly identified as a potential contributor to the association with some certain toxicities.

### Review of Previous Meta-Analysis

As the associations between genetic variations and HDMTX toxicities have become a wide clinical concern, some systematic reviews or meta-analyses have been researched earlier. Initially, we conducted an umbrella review of systematic reviews about the pharmacogenetics of MTX toxicity in patients with osteosarcoma or hematological malignancies in 2019 (Song et al., 2019a). After performing an update search and review in July 2021, we found six similar meta-analyses (Yang et al., 2012; Lopez-Lopez et al., 2013; He et al., 2014; Zhao et al., 2016; Oosterom et al., 2018; Yao et al., 2019) had a discussion on this issue in patients with hematological malignancies. Besides, three meta-analyses (Hagleitner et al., 2014; Zhu et al., 2018; Maagdenberg et al., 2021) included cancer patients and did not distinguish between hematological malignancies and osteosarcoma, although the dosage and infusion regimens of HDMTX vary greatly in the two diseases (Table 4).

**TABLE 4:** Main characteristics of our review and previous meta-analysis

	Present review	Maagdenberg et al. (2021)	Yao et al. (2019)	Oosterom et al. (2018)	Zhu et al. (2018)	Zhao et al. (2016)	He et al. (2014)	Hagleitner et al. (2014)	Lopez-Lopez et al. (2013)	Yang et al. (2012)
Gene	*RFC1*, *SLCO1B1*, *ABCB1*, *GGH*, *FPGS*, *DHFR*, *MTHFR*, *TYMS*, *ATIC*	*RFC1*, *SLCO1B1*, *DHFR*, *MTHFR*, *TYMS*, *MTRR*, *ABCC2*, *andetc*	*MTHFR*	*TYMS*	*MTHFR*	*MTHFR*	*RFC1*	*MTHFR*	*MTHFR*	*MTHFR*
Population	HM^a^	HM, OS^b^	HM	ALL^c^	HM, OS	HM	ALL	HM, OS	ALL	ALL
Age	No restriction	No restriction	No restriction	Pediatric	Pediatric	Adult	Pediatric	No restriction	Pediatric	No restriction
Outcomes	Toxicity, Prognosis	Mucositis	Toxicity, Prognosis	Mucositis	Toxicity	Toxicity	Toxicity	Hepatotoxicity	Toxicity	Toxicity
Dose of MTX	HDMTX	No restriction	No restriction	HDMTX	HDMTX	No restriction	No restriction	No restriction	No restriction	No restriction
HWE restriction	Yes	Yes	No restriction	Yes	No restriction	No restriction	No restriction	No restriction	No restriction	No restriction
Genetic models	Dom^d^,Rec^e^,Alle^f^	Dom, Rec, Alle, Overdom^g^	Dom	Dom	Dom, Rec, Homo^h^	Dom, Rec, Alle, Homo, Het^i^	Dom	Alle	Rec	Dom, Rec, Alle, Homo
Date of search	Dec 2020	Aug 2019	Jan 2018	Oct 2017	Aug 2016	Sep 2015	Sep 2013	Dec 2010	Nov 2011	Sep 2011
Studies included	34	57	17	8	14	6	15	7	24	14

Abbreviation:

a HM: hematological malignancies.

b OS: osteosarcoma.

c ALL: acute lymphoblastic leukemia.

d Dom: dominant model

e Rec: recessive model.

f Alle: allelic model.

g Overdom: overdominant model.

h Homo: homozygote model.

i Het: heterozygote model.

To the best of our knowledge, there have not been any previous meta-analysis to address the following points: 1) investigated 12 genetic polymorphisms within the whole HDMTX pathway and performed analysis on prognosis outcomes, while most previous studies did not discuss these issues (Yang et al., 2012; Lopez-Lopez et al., 2013; Hagleitner et al., 2014; He et al., 2014; Zhao et al., 2016; Oosterom et al., 2018; Zhu et al., 2018); 2) set strict restrictions on hematological malignancies (specified diseases) and HDMTX (specified dose ranges), since the side effect profile of MTX varies markedly as its dose changes in clinical practice; 3) set strict restrictions on the HWE of included pharmacogenetic research, since HWE is crucial for genetic research; 4) analyzed the associations under the dominant model, recessive model, and allelic model, since the genetic models of HDMTX toxicities remain incompletely understood and need validation, whereas most studies only assumed one genetic model; 5) removed restrictions on patient age and ethnicity, which enabled this review to include a greater number of studies and populations than previous studies. The main characteristics of our review and previous meta-analysis are summarized in Table 4. Consequently, our review has provided a comprehensive and up-to-date synthesis of present evidence, and a more reliable conclusion of the association could be reached.

### Biological Mechanisms

Currently, the biological mechanisms linking genetic polymorphisms to HDMTX toxicities still remain incompletely understood. In theory, the delayed MTX clearance or prolonged and elevated exposure of MTX can potentially lead to an increased risk of MTX-induced toxicities. But what is interesting, in one study (Yanagimachi et al., 2013), although an association with higher MTX plasma levels was observed, a genetic association with renal toxicity could not be established, which reminds us that other clinical and genetic factors may play a role together.

The enzyme MTHFR plays a critical role in the folate metabolism by catalyzing the conversion of 5,10-methylene-tetrahydrofolate (THF) to 5-methyl-THF. This is the primary circulating form of folate, which is needed to reduce the toxic homocysteine to methionine. Through this process, folate is an important donor of methyl groups for all intracellular methylation processes (Taylor et al., 2021). The mutation *677C>T* (*rs1801133)* causes a change of alanine to valine in the protein, and the mutation *1298A>C (rs1801131)* causes the replacement of glutamate by valine, resulting in decreased enzyme activity of MTHFR(Robien and Ulrich, 2003). Therefore, the mutations lead to the reduction of folate and then might exert an influence in HDMTX-related toxicities. MTX is extruded by cells using different transporters including ABC family members, and P-glycolprotein (P-gp) is a representative cell membrane protein encoded by the gene *ABCB1* (Castaldo et al., 2011). The *ABCB1* 3435C>T *(rs1045642)* mutations can affect the activity of P-gp and thus may play a role in HDMTX toxicities.

### Limitations and Future Perspective

Several limitations should be considered for our review. First, the outcome measures we investigated were clinical outcomes of HDMTX-related toxicities or prognosis and not the plasma concentration or other pharmacokinetic outcomes of MTX, since the relationships of clinical outcomes and plasma concentration still remain to be verified. And notably, a recent systematic review has discussed on pharmacogenetic factors influencing HDMTX pharmacokinetics (Taylor et al., 2021). Second, data of prognosis outcomes was substantially lacking, which made the quantitative analysis unfortunately impossible for the most prognosis outcomes. Therefore, the genetic associations with prognostic outcomes still remain inconclusive in this study. Third, the sample size of some studies is too small (most are less than 200), and thus, its statistical power might be limited. Furthermore, in addition to the positive findings in the *MTHFR rs1801133* and the *TYMS rs34743033*, statistically significant results of other genetic polymorphisms are only confirmed by meta-analysis of two to three studies. The fact that a meta-analysis of two studies reveals the association between *MTHFR rs1801131* and a decreased risk of renal toxicity (allelic model) means that the trend may not reflect the actual situation, and the results should be cautiously explained. Last but not least, although overall heterogeneity was not observed, the baseline characteristics varied among studies included, including diverse treatment protocols, infusion hours of HDMTX, leucovorin rescue, and other therapy-related factors which might contribute to the associations observed in these studies.

The aforementioned limitations warrant future larger validation studies into genetic association of HDMTX-related clinical outcomes. In line with the research gaps, we would recommend that further studies pay more attention to prognosis outcomes and the genetic polymorphisms of *FPGS (rs10106*, *rs1544105)*, *GGH (rs3758149)*, *DHFR (rs408626*, *rs442767)*, and *ATIC (rs2372536).* And the validation studies are encouraged to calculate the sample size, establish analysis strategies, and prospectively collect data of toxicity and prognosis. With the aim to construct clinically relevant prediction models, the future studies should not focus on the individual effect of single polymorphisms but take into account other polymorphisms within the whole pharmacokinetic and targets/folate pathway. Besides, the prospective cohort studies are encouraged to confirm the clinical benefits of genetic testing by comparing the differences between patients performing genetic testing and those who have not carried out testing.

### Recommendation for Clinical Practice

In light of the findings in this study, associations between genetic variations and the increased/decreased risk of HDMTX toxicities are observed, whereas the association of prognosis outcomes still remains inconclusive due to lacking data. From a clinician or pharmacist’s point of view, we focus more on the role of genetic testing in predicting increased toxicity to tailor MTX therapy, while the data of the potentially protective effect may be less meaningful clinically. With regard to clinical implementation of pharmacogenomics (PGx), the certainty and reliability of supporting evidence are important factors to consider. For instance, the associations between *MTHFR 677C>T (rs1801133)* and an increased risk of hepatotoxicity and mucositis are demonstrated by meta-analysis of nine studies, while the associations between *ABCB1 3435C>T (rs1045642)* and hepatotoxicity are supported by meta-analysis of only three studies.

With the aim to provide practical recommendations, we also reviewed drug instructions of MTX, clinical guidelines or expert consensus of HDMTX, CPIC, and several other pharmacogenetics guidelines. The French National Network of Pharmacogenetics (RNPGx) states that methotrexate pharmacogenetic tests are potentially useful in cancer patients (Quaranta and Thomas, 2017). The evidence-based practice guideline of HDMTX medication of the Chinese Pharmacological Society (Wang et al., 2021) states that the genotyping of *MTHFR 677C>T* and *1298A>C* polymorphisms can be considered for patients with hematological malignancies (weak recommendation, moderate quality evidence), and the genotyping of *ABCB1 3435C>T* may be considered under certain conditions (weak recommendation, low-quality evidence). Further combining the PGx implementation of HDMTX from Chinese perspective (Song et al., 2019b), we would recommend clinicians to consider genetic testing of *MTHFR* polymorphisms when necessary, and *ABCB1* 3435C>T can also be a potential candidate gene. Since patients with gene mutations (*MTHFR 677C>T* particularly) are at the risk of increased hepatotoxicity and/or mucositis, a relatively lower dose and closer monitoring of plasma MTX concentrations are advisable to these patients.

It is worth mentioning that although PGx research has been advanced rapidly in recent years, the clinical implementation of PGx has a long way to go (Guo et al., 2021). To reduce HDMTX-related toxicities and improve outcomes, in addition to the role of genetic polymorphisms, renal function evaluation prior to treatment, co-medications, hydration and urinary alkalization, therapeutic drug monitoring (TDM), and leucovorin rescue might be taken into full consideration. Renal toxicity is one of the most feared side effects of MTX, since the renal dysfunction significantly delays MTX clearance and may cause other toxicities (Schmiegelow, 2009). So far, there is no proven useful approach to predict the individual risk of acute renal failure from the perspective of genetic variation. However, the implementation of standardized hydration and urinary alkalinization and TDM during HDMTX therapy contributes a lot to prevent renal toxicity and maintain MTX elimination.

## Conclusion

In conclusion, the available evidence confirms the associations between the genes *MTHFR* and *ABCB1* and the increased risk of HDMTX toxicity. And a tendency of the genes *RFC1* and *TYMS* toward the decreased toxicity is suggestive in this systematic review. However, current evidence does not support the presence of the associations of the gene *SLCO1B1.* Current studies are often underpowered and unfit to investigate the genetic association of prognosis outcomes. We conclude that genotyping of *MTHFR* and/or *ABCB1* polymorphisms prior to treatment, *MTHFR 677C>T* particularly, is likely to be potentially useful with the aim of tailoring HDMTX therapy and thus reducing toxicity in patients with hematological malignancies. Future larger validation studies into genetic association of HDMTX are still needed.

## Data Availability

The original contributions presented in the study are included in the article/Supplementary Material; further inquiries can be directed to the corresponding author.
