# Supplementary material for: The Role of Genetic Polymorphisms in High-Dose Methotrexate Toxicity and Response in Hematological Malignancies: A Systematic Review and Meta-Analysis
Source: Front Pharmacol. 2021 Oct 21;12:757464. doi: 10.3389/fphar.2021.757464 (PMC8570281; doi:10.3389/fphar.2021.757464)

# Supplemental Materials

## Content


**Supplemental Material I** .......... **3**

Table S1. PRISMA Checklist .......... 3

**Supplementary Material II – Search strategies** .......... **6**

PubMed .......... 6

Embase .......... 6

Cochrane Central Register of Controlled Trials (CENTRAL) .......... 6

Clinical Trials.gov .......... 7

**Supplemental Material III** .......... **8**

Table S2. Quality of cohort studies included in the systematic review and meta-analysis .......... 8

**Supplemental Material IV** .......... **10**

Table S3. Pooled results of the association between *RFC1 (rs1051266)* and HDMTX related outcomes .......... 10

Table S4. Pooled results of the association between *SLCO1B1 (rs4149056)* and HDMTX related outcomes .......... 11

Table S5. Pooled results of the association between *ABCB1 (rs1045642)* and HDMTX related outcomes .......... 12

Table S6. Pooled results of the association between *FPGS (rs10106)* and HDMTX related outcomes .......... 13

Table S7. Meta-analysis results of the association between *FPGS (rs1544105)* and HDMTX related outcomes .......... 13

Table S8. Pooled results of the association between *MTHFR (rs1801133)* and HDMTX related outcomes .......... 14

Table S9. Pooled results of the association between *MTHFR (rs1801131)* and HDMTX related outcomes .......... 16

Table S10. Pooled results of the association between *TYMS (rs34743033)* and HDMTX related outcomes .......... 18

Table S11. Pooled results of the association between *ATIC (rs2372536)* and HDMTX related outcomes .......... 18

**Supplemental Material V** ........................................ **19**

Figure S1. Forest plot for meta-analysis of the association between *RFC1 (rs1051266)* and hepatotoxicity under recessive model ........................................ 19

Figure S2. Forest plot for meta-analysis of the association between ABCB1 (rs1045642) and hepatotoxicity under dominant model ........................................ 20

Figure S3. Forest plot for meta-analysis of the association between *MTHFR (rs1801133)* and hepatotoxicity under dominant model ........................................ 21

Figure S4. Forest plot for meta-analysis of the association between *TYMS (rs34743033)* and mucositis under dominant model ........................................ 22

**Supplemental Material VI** ........................................ **23**

Figure S5. Funnel plot for publication bias test of the association between *MTHFR (rs1801133)* and hepatotoxicity under dominant model ........................................ 23

## Supplemental Material I

## Table S1. PRISMA Checklist

| Section/Topic | Item | Checklist item | Page |
|---|---|---|---|
| **Title** | | | |
| Title | 1 | Identify the report as a systematic review, meta-analysis, or both | P1 |
| **ABSTRACT** | | | |
| Structured summary | 2 | Provide a structured summary including, as applicable, background, objectives, data sources, study eligibility criteria, participants, interventions, study appraisal and synthesis methods, results, limitations, conclusions and implications of key findings, systematic review registration number | P2 |
| **INTRODUCTION** | | | |
| Rationale | 3 | Describe the rationale for the review in the context of what is already known | P3-4 |
| Objectives | 4 | Provide an explicit statement of questions being addressed with reference to participants, interventions, comparisons, outcomes, and study design (PICOS) | P4 |
| **METHODS** | | | |
| Protocol and registration | 5 | Indicate if a review protocol exists, if and where it can be accessed (such as web address), and, if available, provide registration information including registration number | P4 |
| Eligibility criteria | 6 | Specify study characteristics (such as PICOS, length of follow-up) and report characteristics (such as years considered, language, publication status) used as criteria for eligibility, giving rationale | P4-5 |
| Information sources | 7 | Describe all information sources (such as databases with dates of coverage, contact with study authors to identify additional studies) in the search and date last searched | P5 |
| Search | 8 | Present full electronic search strategy for at least one database, including any limits used, such that it could be repeated | Supple II |
| Study selection | 9 | State the process for selecting studies (that is, screening, eligibility, included in systematic review, and, if applicable, included in the meta-analysis) | P5 |
| Data collection process | 10 | Describe method of data extraction from reports (such as piloted forms, independently, in duplicate) and any processes for obtaining and confirming data from investigators | P6 |

| Section/Topic | Item | Checklist item | Page |
|---|---|---|---|
| Data items | 11 | List and define all variables for which data were sought (such as PICOS, funding sources) and any assumptions and simplifications made | P6 |
| Risk of bias in individual studies | 12 | Describe methods used for assessing risk of bias of individual studies (including specification of whether this was done at the study or outcome level), and how this information is to be used in any data synthesis | P6 |
| Summary measures | 13 | State the principal summary measures (such as risk ratio, difference in means) | P6 |
| Synthesis of results | 14 | Describe the methods of handling data and combining results of studies, if done, including measures of consistency (such as $I^2$ statistic) for each meta-analysis | P7 |
| Risk of bias across studies | 15 | Specify any assessment of risk of bias that may affect the cumulative evidence (such as publication bias, selective reporting within studies) | P7 |
| Additional analyses | 16 | Describe methods of additional analyses (such as sensitivity or subgroup analyses, meta-regression), if done, indicating which were pre-specified | P7 |
| **RESULTS** | | | |
| Study selection | 17 | Give numbers of studies screened, assessed for eligibility, and included in the review, with reasons for exclusions at each stage, ideally with a flow diagram | P7 |
| Study characteristics | 18 | For each study, present characteristics for which data were extracted (such as study size, PICOS, follow-up period) and provide the citations | P8 |
| Risk of bias within studies | 19 | Present data on risk of bias of each study and, if available, any outcome-level assessment (see item 12). | P8 |
| Results of individual studies | 20 | For all outcomes considered (benefits or harms), present for each study (a) simple summary data for each intervention group and (b) effect estimates and confidence intervals, ideally with a forest plot | P8-12 |
| Synthesis of results | 21 | Present results of each meta-analysis done, including confidence intervals and measures of consistency | P8-12 |
| Risk of bias across studies | 22 | Present results of any assessment of risk of bias across studies (see item 15) | P12 |
| Additional analysis | 23 | Give results of additional analyses, if done (such as sensitivity or subgroup analyses, meta-regression) (see item 16) | P9-12 |

| Section/Topic | Item | Checklist item | Page |
|---|---|---|---|
| **DISCUSSION** | | | |
| Summary of evidence | 24 | Summarise the main findings including the strength of evidence for each main outcome; consider their relevance to key groups (such as health care providers, users, and policy makers) | P12-13 |
| Limitations | 25 | Discuss limitations at study and outcome level (such as risk of bias), and at review level (such as incomplete retrieval of identified research, reporting bias) | P15-16 |
| Conclusions | 26 | Provide a general interpretation of the results in the context of other evidence, and implications for future research | P17-18 |
| **Funding** | | | |
| Funding | 27 | Describe sources of funding for the systematic review and other support (such as supply of data) and role of funders for the systematic review | P18 |

## Supplementary Material II – Search strategies

## PubMed

**#1** Methotrexate[MeSH] OR Methotrexate[Title/Abstract] OR MTX[Title/Abstract] OR Mexate[Title/Abstract] OR Amethopterin[Title/Abstract]
**#2** "Polymorphism, single nucleotide"[MeSH Terms] OR "Pharmacogenetics"[MeSH Terms] OR "Single nucleotide polymorphism"[Title/Abstract] OR "SNP"[Title/Abstract] OR "Polymorphisms"[Title/Abstract] OR "Pharmacogenetic"[Title/Abstract] OR "Pharmacogenomics"[Title/Abstract] OR "Genetic"[Title/Abstract] OR "Genomic"[Title/Abstract] OR "Genotype"[Title/Abstract] OR "Gene"[Title/Abstract]
**#3** "Hematologic neoplasms"[MeSH] OR "Leukemia"[MeSH] OR "Lymphoma"[MeSH] OR Hematologic neoplasm[Title/Abstract] OR Hematological neoplasm[Title/Abstract] OR Hematologic malignancy[Title/Abstract] OR Hematological malignancy[Title/Abstract] OR Leukemia[Title/Abstract] OR Lymphoma[Title/Abstract]
**#4** Osteosarcoma[MeSH] OR Osteosarcoma[Title/Abstract] OR Sarcoma[Title/Abstract]

**#1 AND #2 AND (#3 OR #4)**
**Filter: Human**
**Up to 2020.11.11**

## Embase

**#1** 'Methotrexate'/exp OR 'Methotrexate':ab,ti OR 'MTX':ab,ti OR 'Mexate':ab,ti OR 'Amethopterin':ab,ti
**#2** 'single nucleotide polymorphism'/exp OR 'pharmacogenetics'/exp OR ‘Single nucleotide polymorphism’:ab,ti OR ‘SNP’:ab,ti OR ‘Polymorphisms’:ab,ti OR ‘Pharmacogenetic’:ab,ti OR ‘Pharmacogenomics’:ab,ti OR ‘Genetic’:ab,ti OR ‘Genomic’:ab,ti OR ‘Genotype’:ab,ti OR ‘Gene’:ab,ti
**#3** 'hematologic malignancy'/exp OR 'leukemia'/exp OR 'lymphoma'/exp OR 'Hematologic neoplasm':ab,ti OR 'Hematological neoplasm':ab,ti OR 'Hematologic malignancy':ab,ti OR 'Hematological malignancy':ab,ti OR 'Leukemia':ab,ti OR 'Lymphoma':ab,ti
**#4** 'osteosarcoma'/exp OR 'Osteosarcoma':ab,ti OR 'Sarcoma':ab,ti

**#1 AND #2 AND (#3 OR #4)**
**Filter: Human**
**Up to 2020.11**

## Cochrane Central Register of Controlled Trials (CENTRAL)

**#1** Methotrexate[MeSH] OR Methotrexate:ti,ab,kw OR MTX:ti,ab,kw OR Mexate:ti,ab,kw OR Amethopterin:ti,ab,kw
**#2** "Polymorphism, single nucleotide"[MeSH Terms] OR "Pharmacogenetics"[MeSH Terms] OR ("Single nucleotide polymorphism" OR "SNP" OR "Polymorphisms" OR "Pharmacogenetic" OR

"Pharmacogenomics" OR "Genetic" OR "Genomic" OR "Genotype" OR "Gene"):ti,ab,kw
**#3** "Hematologic neoplasms"[MeSH] OR "Leukemia"[MeSH] OR "Lymphoma"[MeSH] OR (Hematologic neoplasm OR Hematological neoplasm OR Hematologic malignancy OR Hematological malignancy OR Leukemia OR Lymphoma):ti,ab,kw
**#4** Osteosarcoma[MeSH] OR (Osteosarcoma OR Sarcoma):ti,ab,kw

**#1 AND #2 AND (#3 OR #4)**
**Up to 2020.11.11**

## Clinical Trials.gov

Methotrexate OR MTX | Hematologic Malignancy OR osteosarcoma
**Up to 2020.11.11**

## Supplemental Material III

## Table S2. Quality of cohort studies included in the systematic review and meta-analysis

| Study | Selection of cohorts | | | | Comparability of cohorts | Assessment of outcome | | | Score |
|---|---|---|---|---|---|---|---|---|---|
| | Representativeness of exposed cohort | Selection of non-exposed cohort | Ascertainment of exposure | Outcome was not present at start of study | Comparability | Assessment of outcome | Long enough follow-up | Adequacy of follow-up | |
| Esmaili M A-2020 | * | * | * | | ** | * | | * | 7 |
| Kotur N-2020 | * | * | * | | ** | * | * | | 7 |
| Liu S G-2017 | * | * | * | | ** | * | * | | 7 |
| Den Hoed M-2014 | * | * | * | * | ** | * | * | | 8 |
| Suthandiram S-2014 | * | * | * | | ** | * | * | * | 8 |
| Yanagimachi M-2013 | * | * | * | | ** | | * | | 6 |
| Chiusolo P-2012 | * | * | * | * | ** | * | * | | 8 |
| Faganel K B-2011 | * | * | * | | ** | * | * | * | 8 |
| Faganel K B-2010 | * | * | * | | ** | * | * | * | 8 |
| Ashton L J-2009 | * | * | * | | ** | * | * | * | 8 |
| Imanishi H-2007 | * | * | * | | ** | * | * | * | 8 |
| Shimasaki N-2006 | * | * | * | | ** | * | * | * | 8 |
| Kishi S-2003 | * | * | * | | * | * | * | * | 7 |
| Laverdiere C-2002 | * | * | * | | ** | | * | * | 7 |
| Yang L-2017 | * | * | * | | ** | * | * | * | 8 |
| Avivi I-2014 | * | * | * | * | ** | * | * | * | 9 |
| Fukushima H-2013 | * | * | * | | ** | * | * | * | 8 |
| Tsujimoto S-2016 | * | * | * | | ** | * | * | * | 8 |

| Study | Selection of cohorts | | | | Comparability of cohorts | Assessment of outcome | | | Score |
|---|---|---|---|---|---|---|---|---|---|
| | Representativeness of exposed cohort | Selection of non-exposed cohort | Ascertainment of exposure | Outcome was not present at start of study | Comparability | Assessment of outcome | Long enough follow-up | Adequacy of follow-up | |
| Ma C X-2015 | * | * | * | | ** | * | * | * | 8 |
| Chae H-2020 | * | * | * | | ** | * | * | * | 8 |
| Chang X-2021 | * | * | * | | ** | * | * | * | 8 |
| Giletti-2017 | * | * | * | | ** | * | * | * | 7 |
| Yaz c o-2017 | * | * | * | | ** | * | * | | 7 |
| Choi Y J-2016 | * | * | * | | ** | * | * | * | 8 |
| Erculj N-2014 | * | * | * | | ** | * | * | * | 8 |
| Erculj, N-2012 | * | * | * | | ** | * | * | * | 8 |
| Haase R-2012 | * | * | * | | ** | * | * | | 7 |
| D'Angelo V-2011 | * | * | * | | ** | * | * | | 7 |
| Liu S G-2011 | * | * | * | | ** | * | * | * | 8 |
| Ruiz-Arguelles G J-2007 | * | * | * | | * | | * | * | 6 |
| Seidemann-2006 | * | * | * | | ** | * | * | | 7 |
| Huang Z-2016 | * | * | * | * | ** | * | * | * | 9 |
| Oosterom-2018 | * | * | * | | ** | * | * | | 7 |
| Radtke-2013 | * | * | * | | ** | * | * | * | 8 |

## Supplemental Material IV

## Table S3. Pooled results of the association between *RFC1 (rs1051266)* and HDMTX related outcomes

| *RFC1 A80G (rs1051266)* | MM/Mm vs mm （dominant model) | | | | | MM vs Mm/mm (recessive model) | | | | | M vs m (allelic model) | | | | |
|---|---|---|---|---|---|---|---|---|---|---|---|---|---|---|---|
| | Studies | OR or HR (95%CI) | *Phet* | $I^2$ | P-sub | Studies | OR or HR (95%CI) | *Phet* | $I^2$ | P-sub | Studies | OR or HR (95%CI) | *Phet* | $I^2$ | P-sub |
| Hepatotoxicity | 5 | 0.62 [0.33, 1.16] | 0.02 | 65% | — | **3** | **0.35 [0.16, 0.76]** | **0.91** | **0%** | — | 4 | 0.77 [0.48, 1.21] | 0.68 | 0% | — |
| *Pediatric* | 3 | 0.82 [0.32, 2.07] | 0.01 | 78% | NS [a] | 1 | 0.28 [0.06, 1.38] | NA [b] | NA | NS | 2 | 0.97 [0.51, 1.84] | 0.68 | 0% | NS |
| *Adult* | 2 | 0.49 [0.21, 1.15] | 0.22 | 34% | | **2** | **0.39 [0.16, 0.93]** | **0.79** | **0%** | | 2 | 0.60 [0.31, 1.16] | 0.57 | 0% | |
| **G3-4 hepatotoxicity *(Pediatric)*** | 1 | 0.38 [0.04, 3.29] | NA | NA | — | — | — | — | — | — | — | — | — | — | — |
| **Renal toxicity** | 2 | 0.93 [0.41, 2.10] | 0.88 | 0% | — | 2 | 0.73 [0.21, 2.56] | 0.38 | 0% | — | 2 | 0.91 [0.49, 1.70] | 0.71 | 0% | — |
| *Pediatric* | 1 | 0.96 [0.37, 2.50] | NA | NA | NS | 1 | 1.08 [0.23, 5.00] | NA | NA | NS | 1 | 0.98 [0.48, 2.00] | NA | NA | NS |
| *Adult* | 1 | 0.83 [0.17, 4.04] | NA | NA | | 1 | 0.32 [0.04, 2.91] | NA | NA | | 1 | 0.74 [0.21, 2.64] | NA | NA | |
| **Mucositis** | 4 | 0.91 [0.54, 1.52] | 1 | 0% | — | 3 | 0.99 [0.60, 1.61] | 0.94 | 0% | — | 4 | 0.90 [0.61, 1.32] | 0.61 | 0% | — |
| *Pediatric* | 3 | 0.95 [0.51, 1.74] | 1 | 0% | NS | 2 | 1.03 [0.60, 1.78] | 0.99 | 0% | NS | 3 | 0.93 [0.60, 1.44] | 0.41 | 0% | NS |
| *Adult* | 1 | 0.83 [0.32, 2.15] | NA | NA | | 1 | 0.81 [0.26, 2.55] | NA | NA | | 1 | 0.81 [0.37, 1.79] | NA | NA | |
| **G3-4 Mucositis *(Pediatric)*** | 2 | 1.17 [0.54, 2.52] | 0.23 | 30% | — | 3 | 0.95 [0.53, 1.70] | 0.59 | 0% | — | 2 | 0.97 [0.58, 1.62] | 0.49 | 0% | — |
| **Neurotoxicity *(Pediatric)*** | 2 | 1.22 [0.46, 3.28] | 0.73 | 0% | — | 2 | 1.08 [0.51, 2.27] | 0.71 | 0% | — | 2 | 1.07 [0.58, 2.00] | 0.91 | 0% | — |
| **G3-4 Neurotoxicity *(Pediatric)*** | 2 | 0.52 [0.16, 1.66] | 0.8 | 0% | — | 2 | 0.74 [0.22, 2.50] | 0.38 | 0% | — | 2 | 0.77 [0.31, 1.89] | 0.69 | 0% | — |
| **GI toxicity *(Pediatric)*** | 1 | 0.73 [0.29, 1.79] | NA | NA | — | — | — | — | — | — | 1 | 3.14 [1.08, 9.09] | NA | NA | — |
| Overall toxicity *(Pediatric)* | 1 | 0.65 [0.29, 1.49] | NA | NA | — | **1** | **0.34 [0.12, 0.91]** | **NA** | **NA** | — | 1 | 0.70 [0.39, 1.26] | NA | NA | — |
| **Therapeutic interference *(Pediatric)*** | 1 | 0.94 [0.32, 2.79] | NA | NA | — | 1 | 2.15 [0.63, 7.35] | NA | NA | — | 1 | 1.13 [0.55, 2.34] | NA | NA | — |
| **Relapse *(Pediatric)*** | 1 | 3.52 [0.20, 62.83] | NA | NA | — | 1 | 2.55 [0.56, 11.67] | NA | NA | — | 1 | 1.89 [0.48, 7.43] | NA | NA | — |
| **2y-PFS *(Adult)*** | — | — | — | — | — | **1** | **3.30 [1.21, 9.03]** | **NA** | **NA** | — | — | — | — | — | — |
| **2y-OS *(Adult)*** | — | — | — | — | — | **1** | **2.87 [1.08, 7.65]** | **NA** | **NA** | — | — | — | — | — | — |

| *RFC1 A80G (rs1051266)* | MM/Mm vs mm （dominant model) | | | | | MM vs Mm/mm (recessive model) | | | | | M vs m (allelic model) | | | | |
|---|---|---|---|---|---|---|---|---|---|---|---|---|---|---|---|
| | Studies | OR or HR (95%CI) | *Phet* | I² | P-sub | Studies | OR or HR (95%CI) | *Phet* | I² | P-sub | Studies | OR or HR (95%CI) | *Phet* | I² | P-sub |
| **5y-EFS *(Pediatric)*** | — | — | — | — | — | 2 | 1.43 [0.89, 2.30] | 0.003 | 88% | | — | — | — | — | — |

**Note:** —: No available data

**Abbreviation:** a. NS: No statistical difference; b. NA: Not applicable

# Table S4. Pooled results of the association between *SLCO1B1 (rs4149056)* and HDMTX related outcomes

| *SLCO1B1 T521C (rs4149056)* | MM/Mm vs mm （dominant model) | | | | | MM vs Mm/mm (recessive model) | | | | | M vs m (allelic model) | | | | |
|---|---|---|---|---|---|---|---|---|---|---|---|---|---|---|---|
| | Studies | OR or HR (95%CI) | *Phet* | I² | P-sub | Studies | OR or HR (95%CI) | *Phet* | I² | P-sub | Studies | OR or HR (95%CI) | *Phet* | I² | P-sub |
| **Hepatotoxicity** | 2 | 0.80 [0.44, 1.46] | 0.0008 | 91% | — | — | — | — | — | — | — | — | — | — | — |
| *Pediatric* | **1** | **0.31 [0.13, 0.76]** | **NA** | **NA** | **0.0009*** | — | — | — | — | — | — | — | — | — | — |
| *Adult* | **1** | **3.05 [1.12, 8.32]** | **NA** | **NA** | | — | — | — | — | | — | — | — | — | |
| **Renal toxicity *(Pediatric)*** | 1 | 1.02 [0.36, 2.91] | NA [a] | NA | — | — | — | — | — | — | — | — | — | — | — |
| **G3-4 Mucositis *(Pediatric)*** | 2 | 0.48 [0.22, 1.03] | 0.93 | 0% | — | 1 | 0.56 [0.03, 10.15] | NA | NA | — | 1 | 0.53 [0.20, 1.39] | NA | NA | — |
| **GI toxicity *(Pediatric)*** | — | — | — | — | — | 1 | 1.65 [0.27, 10.02] | NA | NA | — | — | — | — | — | — |
| **Overall toxicity *(Adult)*** | 1 | 0.41 [0.15, 1.15] | NA | NA | — | 1 | 0.11 [0.01, 2.26] | NA | NA | — | 1 | 0.50 [0.19, 1.33] | NA | NA | — |
| **Relapse *(Pediatric)*** | 1 | 1.69 [0.50, 5.68] | NA | NA | — | — | — | — | — | — | — | — | — | — | — |
| **5y-EFS *(Pediatric)*** | — | — | — | — | — | **1** | **12.24 [3.94, 38.07]** | **NA** | **NA** | **—** | — | — | — | — | — |

**Note:** —: No available data

**Abbreviation:** a. NA: Not applicable

## Table S5. Pooled results of the association between *ABCB1 (rs1045642)* and HDMTX related outcomes

| *ABCB1 C3435T (rs1045642)* | MM/Mm vs mm （dominant model) | | | | | MM vs Mm/mm (recessive model) | | | | | M vs m (allelic model) | | | | |
|---|---|---|---|---|---|---|---|---|---|---|---|---|---|---|---|
| | **Studies** | **OR or HR (95%CI)** | ***Phet*** | **$I^2$** | **P-sub** | **Studies** | **OR or HR (95%CI)** | ***Phet*** | **$I^2$** | **P-sub** | **Studies** | **OR or HR (95%CI)** | ***Phet*** | **$I^2$** | **P-sub** |
| **Hepatotoxicity** | **3** | **3.80 [1.68, 8.61]** | **0.29** | **18%** | — | 2 | 1.91 [0.89, 4.09] | 0.17 | 48% | — | 2 | 1.61 [0.89, 2.90] | 0.26 | 21% | — |
| *Pediatric* | 1 | 1.91 [0.60, 6.03] | NA [a] | NA | NS [b] | 1 | 1.11 [0.37, 3.27] | NA | NA | NS | 1 | 1.21 [0.55, 2.63] | NA | NA | NS |
| *Adult* | 2 | 7.46 [2.19, 25.39] | 0.69 | 0% | | 1 | 3.38 [1.07, 10.68] | NA | NA | | 1 | 2.40 [0.97, 5.92] | NA | NA | |
| **Renal toxicity *(Adult)*** | — | — | — | — | — | 1 | 0.71 [0.19, 2.62] | NA | NA | — | — | — | — | — | — |
| **Mucositis** | 3 | 0.82 [0.48, 1.41] | 0.19 | 39% | — | 2 | 0.79 [0.41, 1.54] | 0.24 | 27% | — | 2 | 1.01 [0.63, 1.62] | 0.74 | 0% | — |
| *Pediatric* | 1 | 1.56 [0.57, 4.27] | NA | NA | NS | 1 | 0.53 [0.19, 1.44] | NA | NA | NS | 1 | 0.95 [0.53, 1.71] | NA | NA | NS |
| *Adult* | 2 | 0.60 [0.31, 1.15] | 0.38 | 0% | | 1 | 1.19 [0.47, 3.04] | NA | NA | | 1 | 1.13 [0.50, 2.54] | NA | NA | |
| **G3-4 Mucositis *(Pediatric)*** | 1 | 1.72 [0.86, 3.44] | NA | NA | — | 1 | 1.02 [0.40, 2.59] | NA | NA | — | 1 | 1.01 [0.58, 1.78] | NA | NA | — |
| **Neurotoxicity *(Pediatric)*** | 2 | 0.91 [0.36, 2.33] | 0.89 | 0% | — | 2 | 0.59 [0.24, 1.47] | 0.5 | 0% | — | 1 | 0.81 [0.36, 1.80] | NA | NA | — |
| **GI toxicity *(Adult)*** | 1 | 0.75 [0.36, 1.57] | NA | NA | — | — | — | — | — | — | — | — | — | — | — |
| **Overall toxicity *(Adult)*** | 1 | 0.86 [0.32, 2.31] | NA | NA | — | 1 | 1.63 [0.43, 6.19] | NA | NA | — | 1 | 1.02 [0.47, 2.24] | NA | NA | — |
| **Therapeutic interference *(Pediatric)*** | 1 | 0.80 [0.29, 2.24] | NA | NA | — | 1 | 0.60 [0.21, 1.77] | NA | NA | — | 1 | 0.83 [0.39, 1.76] | NA | NA | — |
| **EFS** | 2 | Esmaili M A-2020: HR>1, P<0.05<br>Ma C X-2015: P=0.076 | | | | | | | | | | | | | |

**Note:** —: No available data

**Abbreviation:** a. NA: Not applicable; b. NS: No statistical difference

## Table S6. Pooled results of the association between *FPGS (rs10106)* and HDMTX related outcomes

| *FPGS A1994G (rs10106)* | MM/Mm vs mm （dominant model) | | | | | MM vs Mm/mm (recessive model) | | | | | M vs m (allelic model) | | | | |
|---|---|---|---|---|---|---|---|---|---|---|---|---|---|---|---|
| | Studies | OR or HR (95%CI) | *Phet* | $I^2$ | P-sub | Studies | OR or HR (95%CI) | *Phet* | $I^2$ | P-sub | Studies | OR or HR (95%CI) | *Phet* | $I^2$ | P-sub |
| Hepatotoxicity *(Adult)* | — | — | — | — | — | 1 | 0.60 [0.27, 1.32] | NA [a] | NA | — | — | — | — | — | — |

**Note:** —: No available data

**Abbreviation:** a. NA: Not applicable

## Table S7. Meta-analysis results of the association between *FPGS (rs1544105)* and HDMTX related outcomes

| *FPGS G2752A (rs1544105)* | MM/Mm vs mm （dominant model) | | | | | MM vs Mm/mm (recessive model) | | | | | M vs m (allelic model) | | | | |
|---|---|---|---|---|---|---|---|---|---|---|---|---|---|---|---|
| | Studies | OR or HR (95%CI) | *Phet* | $I^2$ | P-sub | Studies | OR or HR (95%CI) | *Phet* | $I^2$ | P-sub | Studies | OR or HR (95%CI) | *Phet* | $I^2$ | P-sub |
| 2y-OS *(Adult)* | — | — | — | — | — | **1** | **0.45 [0.24, 0.84]** | **NA [a]** | **NA** | — | — | — | — | — | — |

**Note:** —: No available data

**Abbreviation:** a. NA: Not applicable

# Table S8. Pooled results of the association between *MTHFR (rs1801133)* and HDMTX related outcomes

| *MTHFR C677T (rs1801133)* | MM/Mm vs mm （dominant model) | | | | | MM vs Mm/mm (recessive model) | | | | | M vs m (allelic model) | | | | |
|---|---|---|---|---|---|---|---|---|---|---|---|---|---|---|---|
| | **Studies** | **OR or HR (95%CI)** | ***Phet*** | **$I^2$** | **P-sub** | **Studies** | **OR or HR (95%CI)** | ***Phet*** | **$I^2$** | **P-sub** | **Studies** | **OR or HR (95%CI)** | ***Phet*** | **$I^2$** | **P-sub** |
| **Hepatotoxicity** | **9** | **1.52 [1.03, 2.23]** | **0.01** | **60%** | — | **8** | **1.68 [1.10, 2.55]** | **0.08** | **45%** | — | **7** | **1.41 [1.01, 1.97]** | **0.69** | **0%** | — |
| *Pediatric* | 6 | 1.19 [0.75, 1.89] | 0.05 | 55% | NS [a] | 5 | 0.80 [0.41, 1.54] | 0.34 | 12% | **0.004*** | 4 | 1.02 [0.61, 1.71] | 0.88 | 0% | NS |
| *Adult* | 3 | 2.62 [1.31, 5.25] | 0.07 | 63% | | 3 | 2.76 [1.60, 4.74] | 0.99 | 0% | | 3 | 1.79 [1.15, 2.77] | 0.74 | 0% | |
| **G3-4 Hepatotoxicity** | **3** | **0.16 [0.06, 0.41]** | **0.004** | **82%** | — | 3 | 1.29 [0.57, 2.91] | 0.08 | 45% | — | 2 | 0.89 [0.47, 1.67] | 0.83 | 0% | — |
| *Pediatric* | 2 | 0.74 [0.15, 3.60] | 0.42 | 0% | **0.001*** | 2 | 2.65 [0.56, 11.94] | 0.59 | 0% | NS | 1 | 0.74 [0.12, 4.47] | NA | NA | NS |
| *Adult* | 1 | 0.03 [0.01, 0.10] | NA [b] | NA | | 1 | 0.96 [0.35, 2.63] | NA | NA | | 1 | 0.91 [0.46, 1.79] | NA | NA | |
| **Renal toxicity** | 4 | 1.84 [0.92, 3.69] | 0.07 | 58% | — | **4** | **3.54 [1.81, 6.90]** | **0.1** | **53%** | — | **4** | **1.89 [1.18, 3.02]** | **0.38** | **3%** | — |
| *Pediatric* | 1 | 0.63 [0.24, 1.69] | NA | NA | **0.01*** | 1 | 2.36 [0.23, 24.26] | NA | NA | NS | 1 | 1.67 [0.83, 3.36] | NA | NA | NS |
| *Adult* | 3 | 4.99 [1.48, 16.84] | 0.96 | 0% | | 3 | 3.65 [1.81, 7.36] | 0.04 | 68% | | 3 | 2.09 [1.11, 3.94] | 0.24 | 31% | |
| **G3-4 Renal toxicity *(Pediatric)*** | 1 | 2.33 [0.22, 24.92] | NA | NA | — | — | — | — | — | — | — | — | — | — | — |
| **Mucositis** | **9** | **2.11 [1.31, 3.41]** | **<0.00001** | **79%** | — | 9 | 1.52 [0.95, 2.41] | 0.07 | 46% | — | **8** | **1.91 [1.28, 2.85]** | **<0.00001** | **82%** | — |
| *Pediatric* | 5 | 4.12 [2.28, 7.43] | 0.00 | 78% | **0.0002*** | 5 | 2.46 [1.29, 4.71] | 0.61 | 0% | NS | 4 | 4.60 [2.58, 8.20] | 0.0009 | 82% | **0.0001*** |
| *Adult* | 3 | 0.49 [0.21, 1.14] | 0.18 | 42% | | 3 | 0.79 [0.38, 1.66] | 0.03 | 71% | | 3 | 0.75 [0.41, 1.37] | 0.14 | 49% | |
| *Mixed* | 1 | 7.86 [0.34, 180.33] | NA | NA | | 1 | 1.67 [0.37, 7.61] | NA | NA | | 1 | 1.60 [0.40, 6.36] | NA | NA | |
| **G3-4 Mucositis *(Pediatric)*** | 3 | 1.23 [0.53, 2.85] | 0.29 | 19% | — | 1 | 0.52 [0.02, 11.03] | NA | NA | — | 1 | 0.35 [0.04, 3.37] | NA | NA | — |
| **GI toxicity** | 3 | 0.82 [0.36, 1.87] | 0.57 | 0% | — | 4 | 1.37 [0.59, 3.16] | 0.38 | 2% | — | 3 | 0.89 [0.39, 2.06] | 0.67 | 0% | — |
| *Pediatric* | 2 | 0.63 [0.25, 1.61] | 0.99 | 0% | NS | 2 | 0.91 [0.28, 2.92] | 0.78 | 0% | NS | 2 | 0.60 [0.18, 2.02] | 0.96 | 0% | NS |
| *Adult* | 1 | 2.20 [0.36, 1.87] | NA | NA | | 2 | 2.11 [0.64, 7.03] | 0.16 | 50% | | 1 | 1.28 [0.40, 4.04] | NA | NA | |
| **G3-4 GI toxicity** | 1 | 2.51 [0.10, 66.20] | NA | NA | — | 1 | 1.73 [0.06, 48.17] | NA | NA | — | 1 | 1.56 [0.09, 26.47] | NA | NA | — |

| *MTHFR C677T (rs1801133)* | MM/Mm vs mm （dominant model) | | | | | MM vs Mm/mm (recessive model) | | | | | M vs m (allelic model) | | | | |
|---|---|---|---|---|---|---|---|---|---|---|---|---|---|---|---|
| | Studies | OR or HR (95%CI) | *Phet* | $I^2$ | P-sub | Studies | OR or HR (95%CI) | *Phet* | $I^2$ | P-sub | Studies | OR or HR (95%CI) | *Phet* | $I^2$ | P-sub |
| *(Pediatric)* | | | | | | | | | | | | | | | |
| Dermal toxicity *(Pediatric)* | 1 | 7.82 [0.45, 135.41] | NA | NA | — | 1 | 1.93 [0.58, 6.43] | NA | NA | — | 1 | 1.64 [0.62, 4.32] | NA | NA | — |
| Neurotoxicity *(Pediatric)* | 2 | 0.81 [0.38, 1.73] | 0.14 | 55% | — | 3 | 0.94 [0.29, 3.10] | 0.25 | 29% | — | 1 | 0.40 [0.15, 1.06] | NA | NA | — |
| G3-4 Neurotoxicity *(Pediatric)* | 2 | 0.90 [0.24, 3.40] | 0.53 | 0% | — | 1 | 5.38 [0.30, 95.06] | NA | NA | — | 1 | 0.90 [0.21, 3.77] | NA | NA | — |
| Overall toxicity *(Adult)* | 1 | 0.57 [0.21, 1.58] | NA | NA | — | 1 | 0.85 [0.16, 4.55] | NA | NA | — | 1 | 0.82 [0.38, 1.76] | NA | NA | — |
| Therapeutic interference *(Pediatric)* | **1** | **3.40 [1.31, 8.87]** | **NA** | **NA** | **—** | 1 | 3.79 [0.92, 15.68] | NA | NA | — | 1 | 2.24 [0.97, 5.16] | NA | NA | — |
| Relapse/Death *(Pediatric)* | 2 | 0.81 [0.44, 1.52] | 0.77 | 0% | — | **1** | **3.08 [1.38, 6.88]** | **NA** | **NA** | **—** | 1 | 1.17 [0.65, 2.10] | NA | NA | — |
| 5y-EFS *(Pediatric)* | 2 | 0.83 [0.56, 1.25] | 0.56 | 0% | — | 1 | 0.63 [0.29, 1.35] | NA | NA | — | 2 | Yazicioglu B-2017: CT vs. TT vs. CC: P=0.72<br>Seidemann-2006: CC vs. CT vs. TT: p>0.05 | | | |
| RFS *(Pediatric)* | 1 | 0.70 [0.32, 1.54] | NA | NA | — | — | — | — | — | — | — | — | — | — | — |
| OS *(Pediatric)* | 1 | 1.10 [0.47, 2.57] | NA | NA | — | — | — | — | — | — | — | — | — | — | — |

**Note:** —: No available data

**Abbreviation:** a. NS: No statistical difference; b. NA: Not applicable

# Table S9. Pooled results of the association between *MTHFR (rs1801131)* and HDMTX related outcomes

| *MTHFR A1298C (rs1801131)* | MM/Mm vs mm （dominant model) | | | | | MM vs Mm/mm (recessive model) | | | | | M vs m (allelic model) | | | | |
|---|---|---|---|---|---|---|---|---|---|---|---|---|---|---|---|
| | Studies | OR or HR (95%CI) | *Phet* | $I^2$ | P-sub | Studies | OR or HR (95%CI) | *Phet* | $I^2$ | P-sub | Studies | OR or HR (95%CI) | *Phet* | $I^2$ | P-sub |
| **Hepatotoxicity** | 8 | 0.70 [0.47, 1.03] | 0.29 | 18% | — | 7 | 1.12 [0.61, 2.05] | 0.78 | 0% | — | 5 | 0.88 [0.56, 1.37] | 0.94 | 0% | — |
| *Pediatric* | 6 | 0.59 [0.37, 0.92] | 0.73 | 0% | NS [a] | 5 | 1.29 [0.59, 2.82] | 0.57 | 0% | NS | 3 | 0.75 [0.41, 1.39] | 0.92 | 0% | NS |
| *Adult* | 2 | 1.20 [0.54, 2.66] | 0.6 | 0% | | 2 | 0.92 [0.36, 2.35] | 0.98 | 0% | | 2 | 1.05 [0.55, 1.99] | 0.82 | 0% | |
| **G3-4 Hepatotoxicity *(Pediatric)*** | 2 | 2.89 [0.44, 19.03] | 0.86 | 0% | — | 1 | 2.17 [0.18, 26.29] | NA [b] | NA | — | 1 | 1.63 [0.36, 7.43] | NA | NA | — |
| **Renal toxicity** | 3 | 0.61 [0.32, 1.13] | 0.76 | 0% | — | 2 | 0.79 [0.14, 4.40] | 0.07 | 70% | — | 2 | 0.41 [0.18, 0.97] | 0.59 | 0% | — |
| *Pediatric* | 2 | 0.53 [0.26, 1.11] | 0.75 | 0% | NS | 1 | 5.94 [0.54, 66.05] | NA | NA | NS | 1 | 0.48 [0.18, 1.28] | NA | NA | NS |
| *Adult* | 1 | 0.87 [0.25, 3.04] | NA | NA | | 1 | 0.23 [0.01, 4.43] | NA | NA | | 1 | 0.28 [0.06, 1.45] | NA | NA | |
| **G3-4 Renal toxicity *(Pediatric)*** | 1 | 0.68 [0.09, 5.49] | NA | NA | — | — | — | — | — | — | — | — | — | — | — |
| **Mucositis** | 7 | 0.77 [0.51, 1.17] | 0.76 | 0% | — | 6 | 0.70 [0.33, 1.47] | 0.3 | 18% | | 4 | 0.73 [0.46, 1.15] | 0.98 | 0% | — |
| *Pediatric* | 6 | 0.85 [0.54, 1.35] | 0.79 | 0% | NS | 5 | 0.46 [0.17, 1.28] | 0.32 | 16% | NS | 3 | 0.69 [0.39, 1.21] | 0.95 | 0% | NS |
| *Adult* | 1 | 0.48 [0.18, 1.33] | NA | NA | | 1 | 1.13 [0.38, 3.35] | NA | NA | | 1 | 0.81 [0.37, 1.77] | NA | NA | |
| **G3-4 Mucositis *(Pediatric)*** | 2 | 0.99 [0.18, 5.45] | 0.54 | 0% | — | 1 | 14.00 [1.23, 158.84] | NA | NA | — | 1 | 1.56 [0.20, 12.05] | NA | NA | — |
| **GI toxicity *(Pediatric)*** | 1 | 0.45 [0.02, 11.82] | NA | NA | — | 3 | 0.54 [0.18, 1.64] | 0.7 | 0% | — | 1 | 0.66 [0.04, 11.12] | NA | NA | — |
| **G3-4 GI toxicity *(Pediatric)*** | 1 | 2.23 [0.08, 58.81] | NA | NA | — | 1 | 2.19 [0.08, 62.33] | NA | NA | — | 1 | 1.53 [0.09, 25.90] | NA | NA | — |
| **Dermal toxicity *(Pediatric)*** | 1 | 0.09 [0.01, 1.53] | NA | NA | — | 1 | 1.23 [0.06, 24.20] | NA | NA | — | 1 | 0.12 [0.01, 2.15] | NA | NA | — |
| **Neurotoxicity *(Pediatric)*** | 1 | 1.42 [0.48, 4.22] | NA | NA | — | 2 | 1.36 [0.34, 5.40] | 0.43 | 0% | — | 1 | 1.15 [0.51, 2.60] | NA | NA | — |

| *MTHFR A1298C (rs1801131)* | **MM/Mm vs mm （dominant model)** | | | | | **MM vs Mm/mm (recessive model)** | | | | | **M vs m (allelic model)** | | | | |
|---|---|---|---|---|---|---|---|---|---|---|---|---|---|---|---|
| | **Studies** | **OR or HR (95%CI)** | ***Phet*** | **$I^2$** | **P-sub** | **Studies** | **OR or HR (95%CI)** | ***Phet*** | **$I^2$** | **P-sub** | **Studies** | **OR or HR (95%CI)** | ***Phet*** | **$I^2$** | **P-sub** |
| **G3-4 Neurotoxicity *(Pediatric)*** | 1 | Both are zero-events | NA | NA | — | — | — | — | — | — | — | — | — | — | — |
| **Overall toxicity *(Adult)*** | 1 | 0.93 [0.36, 2.40] | NA | NA | — | 1 | 2.74 [0.27, 27.69] | NA | NA | — | 1 | 1.02 [0.45, 2.30] | NA | NA | — |
| **Therapeutic interference *(Pediatric)*** | 1 | 2.03 [0.79, 5.18] | NA | NA | — | 1 | 1.21 [0.35, 4.18] | NA | NA | — | 1 | 1.41 [0.64, 3.10] | NA | NA | — |
| **Relapse/Death *(Pediatric)*** | 2 | 0.69 [0.36, 1.32] | 0.03 | 79% | — | 1 | 0.78 [0.30, 2.01] | NA | NA | — | 1 | 1.00 [0.58, 1.72] | NA | NA | — |
| **EFS *(Pediatric)*** | 2 | Erculj N-2012: 0.66 [0.33, 1.30]<br>Fukushima H-2013: HR<1, P<0.05 | | | | — | — | — | — | — | 2 | Yazicioglu B-2017: AC vs. CC vs. AA *P=0.59*<br>Radtke S-2013: AC vs AA *HR=1.2 [0.5-2.7] P=0.676*<br>Radtke S-2013: CC vs AA *HR=3.1 [1.2-7.7] P=0.015* | | | |
| **RFS *(Pediatric)*** | 1 | 0.50 [0.22, 1.14] | NA | NA | — | — | — | — | — | — | — | — | | | |
| **OS *(Pediatric)*** | 1 | 0.63 [0.27, 1.45] | NA | NA | — | — | — | — | — | — | — | — | — | — | — |

**Note:** —: No available data

**Abbreviation:** a. NS: No statistical difference; b. NA: Not applicable

## Table S10. Pooled results of the association between *TYMS (rs34743033)* and HDMTX related outcomes

| *TYMS (rs34743033)* | MM/Mm vs mm (dominant model) | | | | | MM vs Mm/mm (recessive model) | | | | | M vs m (allelic model) | | | | |
|---|---|---|---|---|---|---|---|---|---|---|---|---|---|---|---|
| | **Studies** | **OR or HR (95%CI)** | ***Phet*** | **$I^2$** | **P-sub** | **Studies** | **OR or HR (95%CI)** | ***Phet*** | **$I^2$** | **P-sub** | **Studies** | **OR or HR (95%CI)** | ***Phet*** | **$I^2$** | **P-sub** |
| **Hepatotoxicity *(Pediatric)*** | 3 | 0.60 [0.23, 1.59] | 0.75 | 0% | — | — | — | — | — | — | — | — | — | — | — |
| **Renal toxicity *(Pediatric)*** | 1 | 0.87 [0.22, 3.42] | NA [a] | NA | — | — | — | — | — | — | — | — | — | — | — |
| **Mucositis *(Pediatric)*** | 6 | 0.66 [0.47, 0.94] | 0.57 | 0% | — | 1 | 0.90 [0.42, 1.90] | NA | NA | — | 1 | 0.81 [0.40, 1.62] | NA | NA | — |
| **G3-4 Mucositis *(Pediatric)*** | 2 | 0.86 [0.46, 1.60] | 0.04 | 76% | — | 1 | 0.95 [0.33, 2.72] | NA | NA | — | 1 | 1.19 [0.56, 2.52] | NA | NA | — |
| **Neurotoxicity *(Pediatric)*** | 2 | 2.94 [0.55, 15.83] | 0.32 | 0% | — | 1 | 2.16 [0.73, 6.42] | NA | NA | — | 1 | 1.91 [0.67, 5.39] | NA | NA | — |
| **Overall toxicity *(Pediatric)*** | 1 | 0.93 [0.32, 2.74] | NA | NA | — | — | — | — | — | — | — | — | — | — | — |
| **RFS *(Pediatric)*** | 1 | 0.56 [0.24, 1.32] | NA | NA | — | — | — | — | — | — | — | — | — | — | — |
| **OS *(Pediatric)*** | 1 | 0.59 [0.24, 1.45] | NA | NA | — | — | — | — | — | — | — | — | — | — | — |
| **EFS *(Pediatric)*** | 1 | 0.54 [0.26, 1.13] | NA | NA | — | — | — | — | — | — | 1 | Yazicioglu B-2017: 2R3R vs. 3R3R vs. 2R2R: P=0.15 | | | |

**Note:** —: No available data

**Abbreviation:** a. NA: Not applicable

## Table S11. Pooled results of the association between *ATIC (rs2372536)* and HDMTX related outcomes

| *ATIC C347G (rs2372536)* | MM/Mm vs mm (dominant model) | | | | | MM vs Mm/mm (recessive model) | | | | | M vs m (allelic model) | | | | |
|---|---|---|---|---|---|---|---|---|---|---|---|---|---|---|---|
| | **Studies** | **OR or HR (95%CI)** | ***Phet*** | **$I^2$** | **P-sub** | **Studies** | **OR or HR (95%CI)** | ***Phet*** | **$I^2$** | **P-sub** | **Studies** | **OR or HR (95%CI)** | ***Phet*** | **$I^2$** | **P-sub** |
| **Neurotoxicity** | 1 | 1.36 [0.46, 4.06] | NA [a] | NA | — | 1 | 1.74 [0.23, 13.40] | NA [a] | NA | — | — | — | — | — | — |

**Note:** —: No available data

**Abbreviation:** a. NA: Not applicable

## Supplemental Material V

## Figure S1. Forest plot for meta-analysis of the association between *RFC1 (rs1051266)* and hepatotoxicity under recessive model

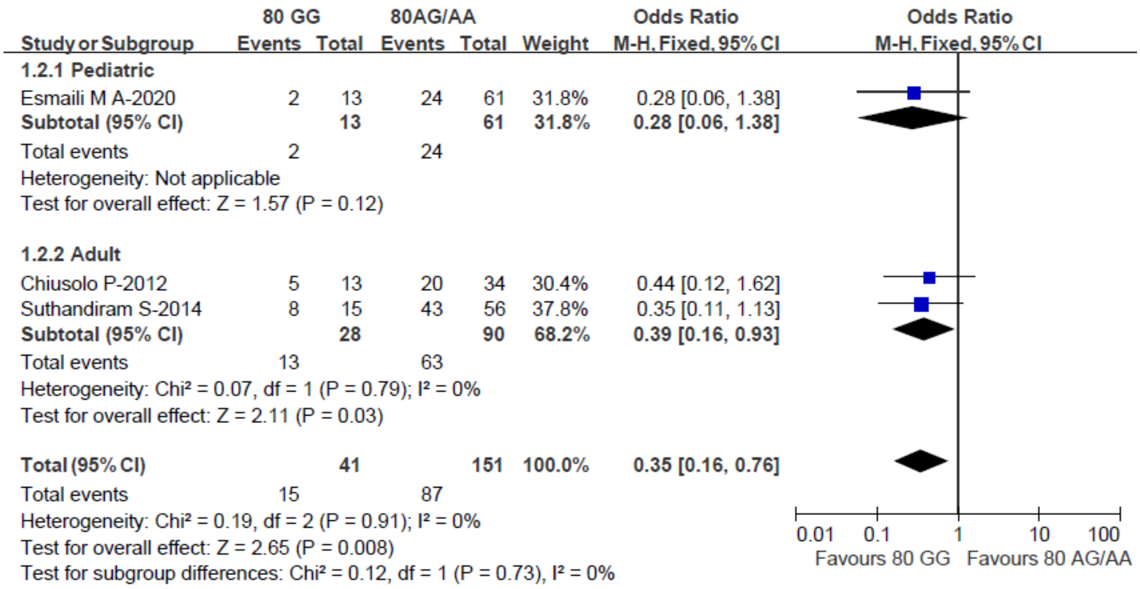

**Figure S2. Forest plot for meta-analysis of the association between *ABCB1* (rs1045642) and hepatotoxicity under dominant model**

| Study or Subgroup | 3435 CT/TT Events | 3435 CT/TT Total | 3435 CC Events | 3435 CC Total | Weight | Odds Ratio M-H, Fixed, 95% CI |
|---|---|---|---|---|---|---|
| **3.1.1 Pediatric** | | | | | | |
| Esmaili M A-2020 | 21 | 54 | 5 | 20 | 65.9% | 1.91 [0.60, 6.03] |
| **Subtotal (95% CI)** | | **54** | | **20** | **65.9%** | **1.91 [0.60, 6.03]** |
| Total events | 21 | | 5 | | | |
| Heterogeneity: Not applicable | | | | | | |
| Test for overall effect: Z = 1.10 (P = 0.27) | | | | | | |
| **3.1.2 Adult** | | | | | | |
| Ma C X-2015 | 10 | 120 | 0 | 58 | 9.1% | 11.12 [0.64, 193.10] |
| Suthandiram S-2014 | 46 | 58 | 5 | 13 | 25.0% | 6.13 [1.70, 22.18] |
| **Subtotal (95% CI)** | | **178** | | **71** | **34.1%** | **7.46 [2.19, 25.39]** |
| Total events | 56 | | 5 | | | |
| Heterogeneity: Chi² = 0.16, df = 1 (P = 0.69); I² = 0% | | | | | | |
| Test for overall effect: Z = 3.22 (P = 0.001) | | | | | | |
| **Total (95% CI)** | | **232** | | **91** | **100.0%** | **3.80 [1.68, 8.61]** |
| Total events | 77 | | 10 | | | |
| Heterogeneity: Chi² = 2.45, df = 2 (P = 0.29); I² = 18% | | | | | | |
| Test for overall effect: Z = 3.20 (P = 0.001) | | | | | | |
| Test for subgroup differences: Chi² = 2.53, df = 1 (P = 0.11), I² = 60.4% | | | | | | |

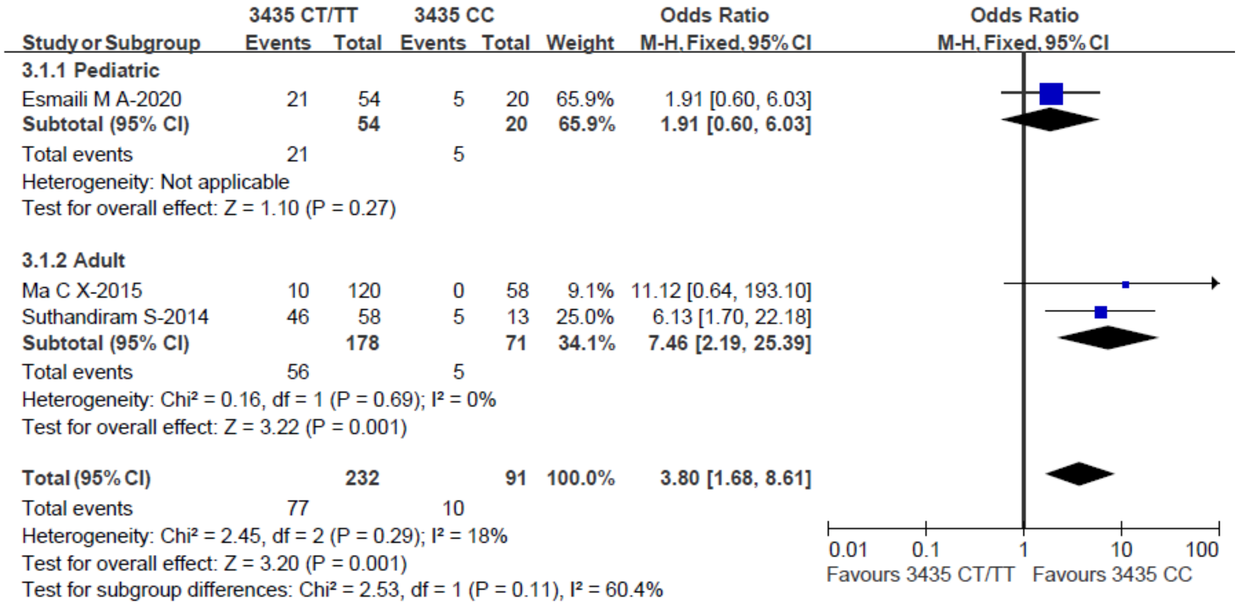

**Figure S3. Forest plot for meta-analysis of the association between *MTHFR (rs1801133)* and hepatotoxicity under dominant model**

| Study or Subgroup | log[Odds Ratio] | SE | Weight | Odds Ratio IV, Fixed, 95% CI |
|---|---|---|---|---|
| **6.1.1 Pediatric** | | | | |
| Erculj N-2014 | -2.1563 | 1.2495 | 2.5% | 0.12 [0.01, 1.34] |
| Haase R-2012 | 0.7538 | 0.9877 | 4.0% | 2.13 [0.31, 14.73] |
| Erculj, N-2012 | -0.3697 | 0.5839 | 11.4% | 0.69 [0.22, 2.17] |
| Liu S G-2011 | -0.2955 | 0.551 | 12.8% | 0.74 [0.25, 2.19] |
| Esmaili M A-2020 | -0.1542 | 0.488 | 16.4% | 0.86 [0.33, 2.23] |
| Fukushima H-2013 | 1.1277 | 0.4212 | 22.0% | 3.09 [1.35, 7.05] |
| **Subtotal (95% CI)** | | | **69.1%** | **1.19 [0.75, 1.89]** |
| Heterogeneity: Chi² = 10.99, df = 5 (P = 0.05); I² = 55% | | | | |
| Test for overall effect: Z = 0.72 (P = 0.47) | | | | |
| **6.1.2 Adult** | | | | |
| Chang X-2021 | 1.9758 | 0.6355 | 9.7% | 7.21 [2.08, 25.06] |
| Suthandiram S-2014 | 1.0627 | 0.6067 | 10.6% | 2.89 [0.88, 9.50] |
| Chiusolo P-2012 | -0.0532 | 0.6044 | 10.7% | 0.95 [0.29, 3.10] |
| **Subtotal (95% CI)** | | | **30.9%** | **2.62 [1.31, 5.25]** |
| Heterogeneity: Chi² = 5.39, df = 2 (P = 0.07); I² = 63% | | | | |
| Test for overall effect: Z = 2.71 (P = 0.007) | | | | |
| **Total (95% CI)** | | | **100.0%** | **1.52 [1.03, 2.23]** |
| Heterogeneity: Chi² = 19.82, df = 8 (P = 0.01); I² = 60% | | | | |
| Test for overall effect: Z = 2.11 (P = 0.04) | | | | |
| Test for subgroup differences: Chi² = 3.43, df = 1 (P = 0.06), I² = 70.8% | | | | |

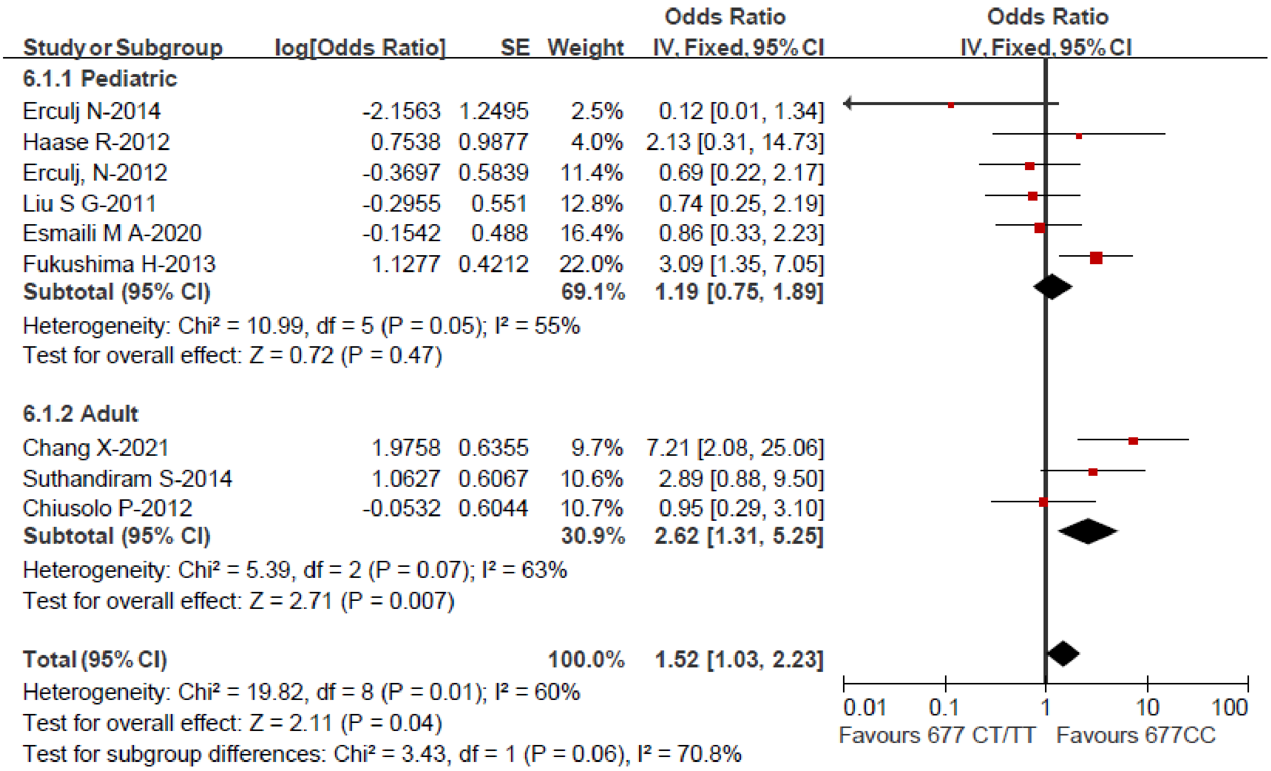

**Figure S4. Forest plot for meta-analysis of the association between *TYMS (rs34743033)* and mucositis under dominant model**

| Study or Subgroup | log[Odds Ratio] | SE | Weight | Odds Ratio IV, Fixed, 95% CI |
|---|---|---|---|---|
| **8.3.1 Pediatric** | | | | |
| Erculj N-2014 | -0.8312 | 0.9327 | 3.6% | 0.44 [0.07, 2.71] |
| Yazicioglu B-2017 | -0.2364 | 0.7046 | 6.2% | 0.79 [0.20, 3.14] |
| Erculj, N-2012 | -0.6048 | 0.5387 | 10.6% | 0.55 [0.19, 1.57] |
| Oosterom N-2017 | 0.3238 | 0.429 | 16.8% | 1.38 [0.60, 3.20] |
| Faganel K B-2011 | -0.5942 | 0.4184 | 17.7% | 0.55 [0.24, 1.25] |
| Radkte S-2013 | -0.5586 | 0.2617 | 45.1% | 0.57 [0.34, 0.96] |
| **Subtotal (95% CI)** | | | **100.0%** | **0.66 [0.47, 0.94]** |
| Heterogeneity: Chi² = 3.84, df = 5 (P = 0.57); I² = 0% | | | | |
| Test for overall effect: Z = 2.34 (P = 0.02) | | | | |
| **Total (95% CI)** | | | **100.0%** | **0.66 [0.47, 0.94]** |
| Heterogeneity: Chi² = 3.84, df = 5 (P = 0.57); I² = 0% | | | | |
| Test for overall effect: Z = 2.34 (P = 0.02) | | | | |
| Test for subgroup differences: Not applicable | | | | |

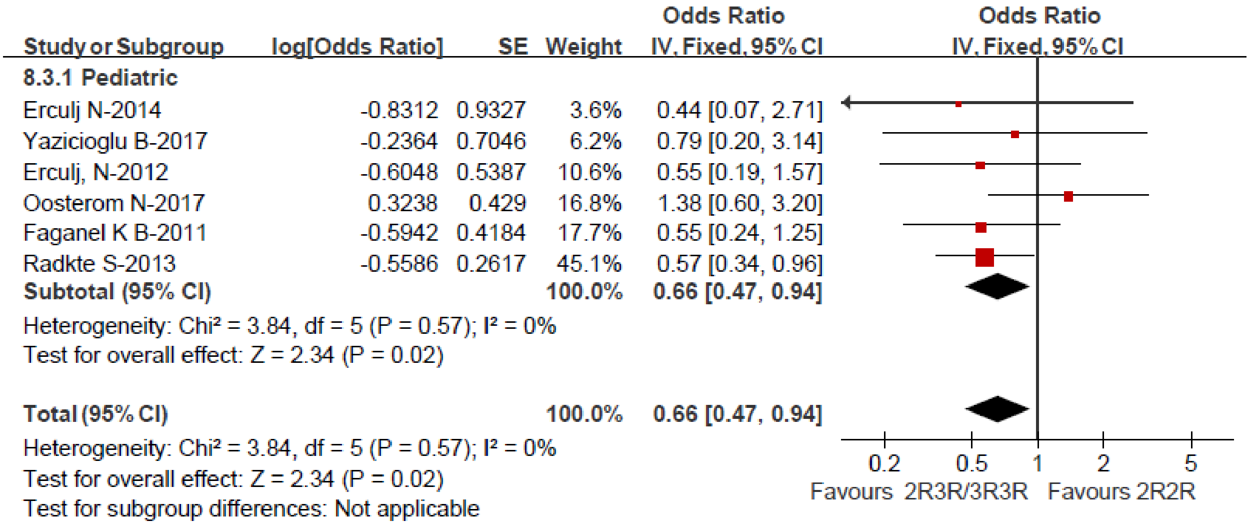

**Supplemental Material VI**

**Figure S5. Funnel plot for publication bias test of the association between *MTHFR (rs1801133)* and hepatotoxicity under dominant model**

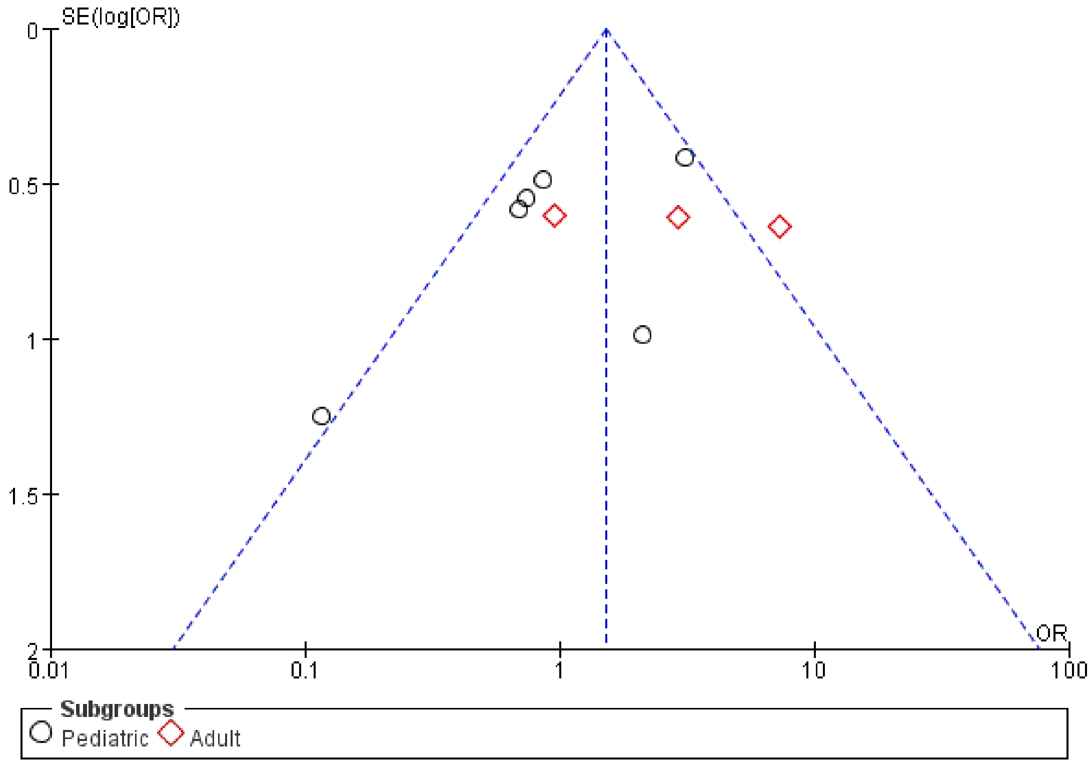

Supplement: Supplementary file 1 [file DataSheet1.pdf]
